# The evolution and polymorphism of mono-amino acid repeats in androgen receptor and their regulatory role in health and disease

**DOI:** 10.3389/fmed.2022.1019803

**Published:** 2022-10-20

**Authors:** Attila Meszaros, Junaid Ahmed, Giorgio Russo, Peter Tompa, Tamas Lazar

**Affiliations:** ^1^VIB-VUB Center for Structural Biology, Vlaams Instituut voor Biotechnologie (VIB), Brussels, Belgium; ^2^Structural Biology Brussels (SBB), Vrije Universiteit Brussel (VUB), Brussels, Belgium; ^3^Research Centre for Natural Sciences (RCNS), ELKH, Budapest, Hungary

**Keywords:** androgen receptor, polyQ, amino acid repeats, polymorphism, phylogenetics, cancer, aggregation, phase separation

## Abstract

Androgen receptor (AR) is a key member of nuclear hormone receptors with the longest intrinsically disordered N-terminal domain (NTD) in its protein family. There are four mono-amino acid repeats (polyQ1, polyQ2, polyG, and polyP) located within its NTD, of which two are polymorphic (polyQ1 and polyG). The length of both polymorphic repeats shows clinically important correlations with disease, especially with cancer and neurodegenerative diseases, as shorter and longer alleles exhibit significant differences in expression, activity and solubility. Importantly, AR has also been shown to undergo condensation in the nucleus by liquid-liquid phase separation, a process highly sensitive to protein solubility and concentration. Nonetheless, in prostate cancer cells, AR variants also partition into transcriptional condensates, which have been shown to alter the expression of target gene products. In this review, we summarize current knowledge on the link between AR repeat polymorphisms and cancer types, including mechanistic explanations and models comprising the relationship between condensate formation, polyQ1 length and transcriptional activity. Moreover, we outline the evolutionary paths of these recently evolved amino acid repeats across mammalian species, and discuss new research directions with potential breakthroughs and controversies in the literature.

## Introduction

**FIGURE 1:**
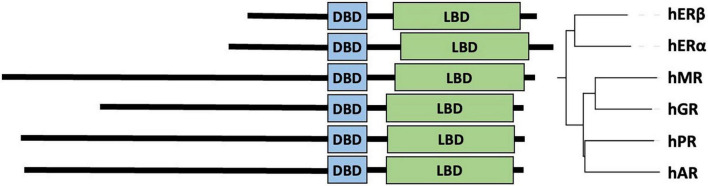
Similarity of type-1 nuclear hormone receptors. Domain architecture aligned by DBD (left) and neighbor-joining phylogenetic tree (right) of six steroid hormone receptor proteins globally aligned (32). UniProt accessions of the sequences (33) are human estrogen receptor beta (hERβ): Q92731, human estrogen receptor alpha (hERα): P03372, human mineralocorticoid receptor (hMR): P08235, human glucocorticoid receptor (hGR): P04150, human progesterone receptor (hPR): P06401, and human androgen receptor (hAR): P10275.

The protein family of nuclear hormone receptors (NHRs) includes several hormone-sensitive transcription factors (TFs), which were discovered and initially characterized as tissue-specific intracellular receptors whose functions are regulated by specific endocrine hormones (1, 2). Sequencing these NHRs [glucocorticoid receptor (GR), estrogen receptor (ER), thyroid hormone receptor, and retinoic acid receptor] revealed a common domain architecture and sequence homology that enabled the establishment of this class as a protein family, and also revealed a large set of orphan receptors with no identified activating hormones (1, 3) and among those mineralocorticoid receptor (MR), androgen receptor (AR), and progesterone receptor (PR) (Figure 1) as reveal later (4–6). The longest isoforms of NHRs generally have a hormone-sensitive ligand-binding domain (LBD), encoded by 5 exons whereas 2 exons encode two zinc-fingers that make up the DNA-binding domain (DBD). In addition, most commonly one exon encodes the N-terminal transactivation domain (NTD) with variable size characteristic to each NHR (Figure 1). In NTD of AR, there is a polymorphic glutamine repeat (polyQ1) and a polymorphic glycine repeat (polyG) with variable length in the human population (see Sections “The polymorphic polyglutamine regions of human androgen receptor” and “The polymorphic polyglycine region of human androgen receptor”), and also in other mammalian species (see Section “Evolution and phylogenetics of the polymorphic repeat regions”). This variability arises due to the slippage of the DNA polymerase during DNA replication caused by the presence of multiple copies of CAG and GGN codons on the template strand (7).

Most NHRs have multiple different isoforms that are shorter than the canonical isoforms. A shorter isoform of AR primarily expressed in castration-resistant prostate cancer (CRPC), ARv7, indicates poorer prognosis in prostate cancer (PCa), as it lacks the LBD and remains overly active even in absence of the hormone, the mechanism of which is still not fully understood (8). Our understanding of these regulatory mechanisms is also limited by the fact that the structure of full-length AR has not been fully determined at near-atomic resolution due to its high degree of flexibility (559 amino acid-long intrinsically disordered NTD) and large size (920 residues). The structures of human AR-LBD (PDB: 4oha) and rat AR-DBD (PDB: 1r4i) have been resolved by X-ray crystallography at 1.42 Å and 3.10 Å resolution, respectively (9, 10). AR-NTD has only been partially modeled (Tau5-R2/3) by nuclear magnetic resonance (NMR) chemical shift-reweighted ensembles (PED: PED00206) based on molecular dynamics simulations (11) and is made available in the Protein Ensemble Database (PED) (12). Recently, the first low-resolution cryo-electron microscopy structure (EMDB: EMD-22079/22080) was reported for transcriptionally active full-length AR, highlighting important conformations in the interdomain cross-talk in AR upon DNA binding and also in complex with Src3 and p300 (13).

In its inactive state, AR localizes in the cytoplasm sequestered by heat shock proteins (HSPs) (13). In a mechanistic structural study, it was shown that Hsp70 and Hsp40 inhibit the NTD-LBD interaction by binding a hydrophobic motif in the NTD (14). Upon binding androgen hormones by the LBD, AR dissociates from HSPs and undergoes homodimerization through LBD and DBD, and enters the nucleus where it binds specific DNA sequences, called androgen response elements (AREs) (13, 15, 16). However, ARv7, which lacks LBD, is constitutively active and resistant against regular cancer treatments that mainly target the LBD. Interestingly, ARv7 also shows constituent nuclear localization despite lacking the natural hinge region, which contains an important nuclear localization sequence (NLS) (17–19). The nuclear shuttling of ARv7 has been shown to occur with a different molecular mechanism compared to the full-length AR (20). In the NTD, there have been important regions proposed to regulate the efficient transcription activation function, including a transactivation domain AF1 (aa. 142-485). AF1 is responsible for recruiting different partners and co-factors to regulate transcription, such as SRC1, SRC3, p300, TFIIF via various motifs (21–23). Moreover, the highly conserved first 30 residue of NTD contains a hydrophobic binding motif (FQNLF), which interacts with the LBD or melanoma-associated antigen-11 (MAGE-A11), thereby regulating the NTD-LBD interaction (24). The same hydrophobic motif is responsible for binding Hsp40 and Hsp70 in the cytosol (14). Lastly, the length-polymorphic glutamine and glycine stretches (polyQ1/polyG) have also been shown to affect the transcriptional activity (25–27).

Intriguingly, besides physiological cytoplasmic sequestering, HSPs can also affect AR signaling in PCa by triggering the degradation of the receptor, thereby resulting in lower transactivation (28, 29). Even though ARv7 seemed to be resistant to first-generation HSP inhibitors (28, 30), a new, second-generation HSP90 inhibitor decreased ARv7 level through a different mechanism, affecting the mRNA splicing of this variant (31).

In this review, we initially focus on the structure-function relationship of AR’s polymorphic (polyQ1, polyG) and non-polymorphic (polyQ2, polyP) mono-amino acid repeat regions, the evolution and phylogenetic differences thereof, also highlighting current controversies in the literature. In the end, we list a few well-known and recently identified research gaps and propose future research directions with high potential for great breakthroughs.

## Structure, function, and disease relevance of the androgen receptor’s mono-amino acid repeat regions

### The polymorphic polyglutamine regions of human androgen receptor

Polyglutamine repeat tracts are frequent in the proteome from yeast to humans and they are over-represented in the activation domain of TFs (34). In yeast, even small modification in the number of glutamine residues in the polyQ region of the transcription factor Ssn6 (Cyc8) resulted in phenotypic differences between strains as well as different fitness under certain nutrient stress (34). The exact mechanism of action is still under debate. However, the most accepted explanation is that the length of the polyQ influences the solubility and conformation of the protein (35). Therefore, a difference in polyQ length can alter the interaction of the TF with its cofactors, hence affecting upregulation or downregulation of target genes.

The transactivation domains of TFs are usually intrinsically disordered and considered to be of low complexity (36–39). The NTD of AR has also been predicted to be disordered by all the prediction tools available, which has been also verified experimentally (40, 41). However, a study based on circular dichroism and fluorescence emission spectra suggested that the polyQ1 region has an alpha-helical propensity (42), which was later verified by *in vitro* NMR experiments. In this NMR study of the first 153 residues of the NTD, it was shown that the polyQ1 stretch has alpha-helical structure, while the rest of the sequence displayed no persistent secondary structure (43). Interestingly, when deleting the four leucines (55-LLLL-58) preceding polyQ1, helix formation was disrupted and the aggregation propensity of the construct highly increased (43). This study also revealed that the length of the polyQ1 correlates with the aggregation propensity of the fragment. Due to this, the authors concluded that the longest polyQ1 fragment that can be studied *in vitro* is 25Q. In a follow-up NMR study, the same group showed that the side chains of glutamines form H-bonds with the main chain (44). The strength of these H-bonds is determined by the H-bond acceptor, leucines being better acceptors than alanines, providing an explanation to the conservation of leucines preceding the polyQ1. Interestingly, in the case of polyQ2, there are two leucines, which also show a high level of conversation in mammals (see Section “Evolution and phylogenetics of the polymorphic repeat regions”). It is important to mention here that the polyQ region of huntingtin in Huntington’s disease also has an important polyP flanking region, which decreases aggregation propensity (45), highlighting the potential role of solubility-enhancing flanking regions as an evolutionary mechanism to mitigate cytotoxicity. In addition, a study in yeast also found that the flanking regions of polyQ repeats can profoundly alter their toxicity (45, 46).

In humans, nine proteins with polymorphic polyQ repeats–including AR–have pathological implications when their repeat lengths are out of the physiological range (47, 48). These proteins have been the subject of various studies to shed light on the molecular mechanism of the relationships between the length and biological effect (Table 1A). In the case of AR, there is a physiological range between 9 and 36 (26, 49). PolyQ1 stretches longer than 37 successive Qs have been reported to form neurotoxic aggregates (50), as according to the proposed mechanism, longer Q-repeats decrease solubility and hence allow for fibrillar aggregate formation (51, 52), leading to a disease called spinal-bulbar muscular atrophy (SBMA), also known as Kennedy’s disease (53). In case of the disease, the patients show androgen insensitivity worsening with age, typically affecting adult males at older age (54). Affected patients have an expanded polyQ1 tract between 38 and 62 glutamines (55) and similarly to other CAG repeat expansion related diseases, the length of expansion is inversely correlated with the age of onset, disease severity and progression (55, 56). There are multiple pathways along the transformation from physiological to pathological state. Due to the previously mentioned aggregation tendency of the expanded polyQ1 region, there is a gain-of-function toxicity that results in the loss of alteration of normal AR function (57). Moreover, the elimination of the misfolded AR is hindered by autophagy dysregulation (58). Lim and et al. (59) have recently shown that delivering a naturally occurring AR isoform–isoform 2 that lacks the polyQ1 harboring NTD–by adenovirus vector can rescue the neurotoxic phenotype in SBMA mice models. This provided proof-of-principle type of evidence of the role of AR with extended polyQ1 in disease, and a possible future therapeutic approach by gene therapy.

**TABLE 1:** CAG and GGN repeat ranges and distribution peaks across different cohorts.

Cohort	*N*	Min. repeats	Max. repeats	Distrib. peak	Source
**(A) CAG repeats**					
Caucasian men (healthy)	39	17	27	20	Irvine et al. (98)
African-American men (healthy)	44	9	27	17 and 22	Irvine et al. (98)
Asian men (healthy)	39	15	29	21–22	Irvine et al. (98)
Asian men (healthy)	305	14	32	23	Kawasaki et al. (97)
Asian men (rheumatoid ar.)	90	12	30	21	Kawasaki et al. (97)
Asian women (healthy)	332*	12	31	23	Kawasaki et al. (97)
Asian women (rheumatoid ar.)	226*	12	30	23	Kawasaki et al. (97)
African-American men (healthy)	516	9	31	15	Kittles et al. (74)
Sierra Leonean men (healthy)	230	10	26	16	Kittles et al. (74)
Nigerian men (healthy)	83	5	28	16	Kittles et al. (74)
Caucasian men (healthy)	87	13	26	20	Kittles et al. (74)
Amerindian mean (healthy)	80	14	30	22	Kittles et al. (74)
Asian men (healthy)	60	14	26	20	Kittles et al. (74)
Caucasian men (healthy)	370	7	31	21	Hakimi et al. (99)
Caucasian men (PCa)	59	16	29	20	Hakimi et al. (99)
Caucasian men (healthy)	390	13	30	21	Edwards et al. (100)
Caucasian men (PCa)	178	14	32	21	Edwards et al. (100)
Caucasian men (healthy)	67	14	31	21	Binnie et al. (101)
Caucasian men (BPH)	77	8	36	24–25	Binnie et al. (101)
Caucasian men (PCa)	100	8	32	21	Binnie et al. (101)
Caucasian men (healthy)	115	9	31	23	Ferlin et al. (89)
Caucasian men (infertile)	163	9	29	21	Ferlin et al. (89)
Caucasian men (healthy)	446	12	35	22	Freedman et al. (102)
Caucasian men (PCa)	405	12	34	21	Freedman et al. (102)
African-American men (healthy)	664	11	41	20	Freedman et al. (102)
African-American men (PCa)	637	11	33	19	Freedman et al. (102)
Asian men (healthy)	476	12	33	23	Freedman et al. (102)
Asian men (PCa)	431	12	38	23	Freedman et al. (102)
Hispanic men (healthy)	574	12	38	23	Freedman et al. (102)
Hispanic men (PCa)	576	12	33	23	Freedman et al. (102)
Caucasian men (healthy)	60	15	27	23	delli Muti et al. (103)
Caucasian men (infertile)	40	18	31	22	delli Muti et al. (103)
Caucasian men (healthy)	974	13	33	22	Grigorova et al. (104)
Caucasian† women (healthy)	461*	8	38	21	Suter et al. (105)
Caucasian† women (BRCa)	524*	8	35	21	Suter et al. (105)
Caucasian women (healthy)	461*	n.a.	n.a.	21	Giguere et al. (106)
Caucasian women (BRCa)	255*	11	32	21	Giguere et al. (106)
Caucasian women (healthy)	430	11	36	21	Gonzalez et al. (107)
Caucasian women (BRCa)	300	13	37	21	Gonzalez et al. (107)
Caucasian women (ECa)	207*	12	32	21	Rodriguez et al. (108)
Caucasian men (healthy)	106	13	29	21	Rodriguez-Gonzalez et al. (109)
Caucasian men (PCa)	72	16	29	21	Rodriguez-Gonzalez et al. (109)
Caucasian men (healthy)	141	15	34	22	Westberg et al. (110)
Caucasian men (violent criminals)	63	12	29	21	Westberg et al. (110)
Caucasian men (healthy)	422	11	31	mean: 21.9	O’Brien et al. (111)
African-American men (healthy)	150	11	29	mean: 19.8	O’Brien et al. (111)
African men (healthy)	112	12	31	mean: 20.0	O’Brien et al. (111)
Hispanic mean (healthy)	63	21	29	mean: 25.1	O’Brien et al. (111)
African-American men (healthy)	340	9	29	18 and 21	Lange et al. (112)
African-American men (PCa)	130	10	30	18 and 22	Lange et al. (112)
African men (healthy)	123	<14	>25	20 and 22	Akinloye et al. (113)
African men (BPH)	68	<14	24	22	Akinloye et al. (113)
African men (PCa)	70	<14	24	22	Akinloye et al. (113)
Caucasian men (healthy)	557	6	37	23	Brokken et al. (114)
Greenlander men (healthy)	213	13	29	24	Brokken et al. (114)
Caucasian women (healthy)	4421*	13	32	21	Ackerman et al. (68)
Asian women (healthy)	1494*	13	32	22	Ackerman et al. (68)
Afro-Caribbean women (healthy)	1119*	13	31	18	Ackerman et al. (68)
Hispanic women (healthy)	780*	13	32	23	Ackerman et al. (68)
Caucasian men and women (SBMA)	159	40	54	45	Bertolin et al. (115)
Diverse group of men and women (SBMA)	35	40	52	n.a.	La Spada et al. (53)
Diverse group of men (healthy)	213	14	35	21	Macke et al. (116)
Brazilian# men (healthy)	279	11	30	mean: 22.1	dos Santos et al. (117)
Brazilian# men (PCa)	133	14	30	mean: 21.8	dos Santos et al. (117)
Asian men (healthy)	300	10	33	23	Hsing et al. (118)
Asian men (PCa)	190	15	34	22	Hsing et al. (118)
Asian men (healthy)	104	15	31	mean: 22.9	Huang et al. (119)
Asian men (PCa)	66	15	31	mean: 23.2	Huang et al. (119)
Asian men and women (healthy)	449	11	37	22	Kovacs et al. (120)
**(B) GGN repeats**					
Caucasian men (healthy)	37	16	23	22	Irvine et al. (98)
African-American men (healthy)	41	14	23	21	Irvine et al. (98)
Asian men (healthy)	37	16	23	22	Irvine et al. (98)
Caucasian men (healthy)	794	10	29	23	Platz et al. (121)
Caucasian men (PCa)	582	14	28	23	Platz et al. (121)
African-American men (healthy)	472	10	26	20	Kittles et al. (74)
Sierra Leonean men (healthy)	210	10	30	20	Kittles et al. (74)
Nigerian men (healthy)	78	14	25	20	Kittles et al. (74)
Caucasian men (healthy)	80	8	26	21	Kittles et al. (74)
Amerindian mean (healthy)	103	14	22	21	Kittles et al. (74)
Asian men (healthy)	60	16	22	21	Kittles et al. (74)
Caucasian men (healthy)	588	6	39	21	Giovannucci et al. (122)
Caucasian men (PCa)	587	12	35	21	Giovannucci et al. (122)
Caucasian men (healthy)	370	16	24	22	Hakimi et al. (99)
Caucasian men (PCa)	59	16	28	22	Hakimi et al. (99)
Caucasian men (healthy)	284	13	17	22	Edwards et al. (100)
Caucasian men (PCa)	178	15	26	22	Edwards et al. (100)
Caucasian men (healthy)	67	9	28	22	Binnie et al. (101)
Caucasian men (BPH)	77	15	29	22–23	Binnie et al. (101)
Caucasian men (PCa)	100	8	27	22	Binnie et al. (101)
Caucasian men (healthy)	115	14	27	23	Ferlin et al. (89)
Caucasian men (infertile)	163	10	28	23	Ferlin et al. (89)
Caucasian men (healthy)	60	23	29	24	Muti et al. (103)
Caucasian men (infertile)	40	17	26	23	Muti et al. (103)
Caucasian men (healthy)	974	10	28	23	Grigorova et al. (104)
Caucasian† women (healthy)	443*	9	24	22	Suter et al. (105)
Caucasian† women (BRCa)	515*	9	24	22	Suter et al. (105)
Caucasian women (healthy)	430	12	30	23	Gonzalez et al. (107)
Caucasian women (BRCa)	300	12	30	23	Gonzalez et al. (107)
Caucasian women (ECa)	207*	13	27	22	Rodriguez et al. (108)
Caucasian men (healthy)	106	13	27	23	Rodriguez-Gonzalez et al. (109)
Caucasian men (PCa)	72	17	25	23	Rodriguez-Gonzalez et al. (109)
Caucasian men (healthy)	141	19	27	23	Westberg et al. (110)
Caucasian men (violent criminals)	63	17	29	23	Westberg et al. (110)
Caucasian men and women (SBMA)	159	20	24	23	Bertolin et al. (115)
African-American men (healthy)	340	5	21	17	Lange et al. (112)
African-American men (PCa)	129	7	21	17	Lange et al. (112)
African men (healthy)	123	<14	>25	21	Akinloye et al. (113)
African men (BPH)	68	<14	23	21	Akinloye et al. (113)
African men (PCa)	70	16	>25	21	Akinloye et al. (113)
Caucasian men (healthy)	557	10	30	23	Brokken et al. (114)
Greenlander men (healthy)	213	20	27	23	Brokken et al. (114)
Diverse group of men (healthy)	213	12	30	23	Macke et al. (116)
Chinese men (healthy)	295	14	27	23	Hsing et al. (118)
Chinese men (PCa)	187	15	25	23	Hsing et al. (118)
Chinese men and women (healthy)	449	17	27	23	Kovacs et al. (120)
Japanese men (healthy)	102	16	24	22	Sasaki et al. (123)
Japanese women (healthy)	200*	16	23	22	Sasaki et al. (123)
Japanese women (ECa)	226*	16	27	22	Sasaki et al. (123)

*As women have 2X chromosomes, the real sample size of AR alleles genotyped was two times higher.

^†^89% Caucasian, 5% Asian, 4% African American, 1% American Indian, 1% Hispanic.

^#^73% White Brazilian, 25% Afro-Brazilian, 3% Asian origin. PCa, prostate cancer; BPH, benign prostate hyperplasia; BRCa, breast cancer; ECa, endometrial cancer; SBMA, spinal-bulbar muscular atrophy.

In addition to the intrinsic aggregation propensity of the polyQ1, a short, highly conserved sequence upstream from this repeat (235-KELCKAVSVSM-245) has recently been reported to undergo reversible amyloid fiber formation under mild oxidative conditions (60). In a follow-up study, the same group showed by atomic force microscopy that the oligomeric state of AR-NTD fragment was modulated by this amyloidogenic sequence, suggesting that this region can function as a nucleation center for subsequent aggregation of polyQ1 (61). However, they did not observe fibril formation for a polyQ of a length within the physiological range (22), only in case of aberrant length (45). Interestingly, this region partially overlaps with the binding site of the RNA polymerase-associated protein 74 subunit of the general transcription factor TFIIF, and mutation of conserved bulky hydrophobic residues in this sequence to smaller hydrophobic alanine significantly impaired transcriptional activity (62). Moreover, EPI-001, a compound that binds specifically to AR-NTD inhibiting transcriptional activity, showed weak chemical shift perturbation in this region by NMR titration (63). In this and a follow-up NMR study intermediate helical propensity was observed in this region (23, 63).

It is important to note that polyQ1 displays not only an increased aggregation propensity upon pathological expansion (64), it also has a negative correlation with transcriptional activity of AR (65–67). It has been suggested that the length of polyQ1 influences NTD–LBD interaction, which can affect the activity of AR (26). There is also a difference in the polyQ1 length among ethnicities (Table 1A): African people have the shortest, Asian people the longest and Caucasian people in between (68). Furthermore, shorter repeats are associated with higher PCa propensity (25, 69, 70). The commonly accepted hypothesis is that the length of the polyQ1 and the transcriptional activity of AR are inversely correlated, and long-term exposure of prostate cells to elevated AR activity can increase proliferation and trigger oncogenic transformation. This further supports the argument that males with African ancestry have shorter CAG repeats on average (Table 1A) in comparison to non-hispanic Caucasian and Asian people (71–74) and have higher mortality caused by PCa than Caucasian and Asian people (75–77). However, detailed comparison of these studies led us to pinpoint various controversies, which we are going to dissect in more detail in Section “Controversies.” In addition, a recent review summarizes additional biological risk factors based on new genome-wide association studies as well as environmental and social risk factors with regards to African or European ancestry (78).

It has been well-established in the literature that Wnt signaling pathway is often misregulated in disease, especially in cancers like PCa, where it drives oncogenic proliferation (79, 80). Elevated β-catenin expression in the nucleus enhances tumorigenesis in the prostate (81–83) promoting a very aggressive form of PCa with poor survival (84). Conversely, in patients with early onset PCa with very severe tumor growth (84, 85) and in many African American PCa patients (86), both Wnt and androgen signaling are significantly upregulated. In a recent study, He et al. (87) highlighted the involvement of polyQ1 in this misregulation. Using compound mice with humanized AR sequence bearing different polyQ1 lengths (12, 21, and 48 glutamines), they found that short polyQ1–compared to the longer counterpart–displayed an earlier onset of oncogenic transformation along with accelerated and more aggressive tumor development in the prostate. These results provide further explanation to the already existing hypothesis (i.e., short polyQ1 results in higher activity) and highlight the complexity of PCa tumorigenesis.

Androgen receptor activity is essential for spermatogenesis (88), however, most men with aberrant spermatogenesis have normal serum androgen levels (66). Therefore, researchers explored the possible involvement of the polymorphic regions of AR, as they can modulate AR level and activity independently of the androgen serum level (as mentioned before). In an early study, Tut et al. (66) analyzed samples from patients (*N* = 153) with normal androgen serum level, and found a significant correlation between the length of polyQ1 and defective sperm production. They found that longer polyQ1 repeats (≥28) increased the risk of impaired spermatogenesis four fold. However, later many more studies came to conflicting results, some showing correlation while others don’t (89). Ferlin et al. (89) also failed to confirm the link and only observed some association when the two polymorphic regions polyQ1 and polyG were analyzed separately, and only in the case of a few individuals (Table 1). It is important to mention that most of the studies were performed in different parts of the world and usually on a subset of the local population.

Because COVID-19-associated intensive care admission as well as mortality is higher in men than in women, researchers started to explore the possible explanations (90, 91). AR regulates the transcription of transmembrane protease serine 2 (TMPRSS2) (92), which primes the spike protein of the virus, therefore the spike can bind to the receptor of the host cell and enter (93). Mohamed et al. (94) in a recent review proposed that shorter CAG repeats confer higher AR activity, therefore higher TMPRSS2 transcription which causes higher risk of severe disease outcome. However, later, two independent studies with patient samples came to similar conclusions arguing for opposing trends. One of these studies observed that European males from Italy and Spain with longer CAG repeat (≥23) had worse clinical outcomes due to severe COVID-19 than patients with shorter CAG repeat (≤22) (95). The other study also concluded that longer CAG repeat (≥22) has conferred worse COVID-19 outcome in males (96).

Finally, another disease-linked correlation regarding the length of the polyQ1 was reported by Kawasaki et al. (97) who found that short polyQ increases the risk of early onset rheumatoid arthritis in males younger than 55.

### The polymorphic polyglycine region of human androgen receptor

The polymorphic polyglycine (polyG) region is also located within the intrinsically disordered NTD of AR, and is encoded by three GGT triplets followed by one GGG and two more GGT triplets, and a variable number of GGC triplets. It has been shown that the length of this region has an effect on the translation of the protein itself (124, 125) and potentially also on the transcriptional activity of AR (27, 125, 126). For example, recombinantly expressed AR constructs with only 10 GGN repeats decreased relative AR activity to 40–68% of the wild-type (27, 126), while longer GGN repeats with a glycine stretch of 27 also exhibited reduced activity of 37–78% (126). In case of genetically engineered AR constructs with shorter and longer GGC repeats, protein abundance was found to be inversely correlated with polyG length, and it is hypothesized that the longer GGC repeats form a more stable hairpin structure in the mRNA that interferes more with translation (124, 125).

Moreover, it has been established that across certain races and ethnic groups the range of polyG/GGN repeat variation exhibits differences (Table 1B), and hence, this factor has to be considered during study design. In the following, we summarize the most significant findings on the relationship between diseases and polyG length.

In light of the molecular details mentioned above, it is not surprising that the polymorphic length of GGN repeats, and consequently the polyG tract, is a risk factor in certain cancer types (99, 123, 127) correlating with progression and/or severity of the disease, or the outlook for relapse-free periods (100, 108, 109). However, it is important to note that there are still significant controversies in the literature on the importance of the polyG length in particular cancer types (see the “Controversies” Section for details and Table 1B).

In an early study, Hakimi et al. (99) found that both polyG ≤ 14 and polyQ ≤ 17 are more common in the general Caucasian male population with clinical PCa diagnosis (*N* = 59), and patients with any of the two allele types have higher odds of developing malignancy, although the frequencies of the polymorphisms seem to be independent of each other. A large meta-analysis on the relationship between PCa and AR polymorphisms in the Caucasian population showed that short polyG of max. 16 repeats imposed the same amount of risk for PCa than a short polyQ with less than 22 repeats, while the combination of both short polyQ and polyG doubles the odds ratio of PCa risk (95% CI: 1.29–3.29) (127). Edwards et al. (100) found that long GGC repeats of more than 16 significantly increased the risk of relapse and risk of death in British Caucasian men (*N* = 178) from around 33 months after PCa diagnosis; furthermore, long GGC repeats were associated with a worse prognosis and survival at all disease levels of stage and grade. In men from the Canary Islands (*N* = 72), an immunohistochemistry study showed that the polyG length was negatively correlated to prostate specific antigen (PSA) staining intensity, especially in samples with simultaneously shorter polyQ or from the more severe type of PCa with Gleason score of at least 7 (109).

On the other hand, longer polyG repeats also come with a risk for women (Table 1B): Based on a study on a Japanese cohort (*N* = 226), longer GGC repeats (≥17 GGC) are more frequently associated with endometrial cancer (ECa) as compared to the control population (123). In women from the Canary Islands (*N* = 207), shorter polyG was found to be more frequently associated with benign type of ECa with slower cancer progression and better outcomes (108). In a cohort from the USA with 89% Caucasian study participants, longer GGC repeats were associated with reduced risks of breast cancer (BRCa) (105, 108). Gonzalez et al. found that the combination of long polyQ (>22) and long polyG (≥24) is more common in female BRCa patients from the Canary Islands (*N* = 257) than average polyQ (>22) (105, 107, 108).

As AR has a key role in androgen insensitivity syndrome (AIS), a disease often leading to defects in virilization and infertility, investigating the role of mutations, including the polymorphic GGN/polyG alleles, are of high importance. Grigorova et al. (104) found that those with decreased sperm counts more commonly had longer GGN repeats. Although polyG length alone was not found to be prognostic to infertility, it may further tune the effects of other mutations or polymorphisms. In accord, lowest sperm counts were found in individuals with both longer GGN and longer CAG repeats. Another study carried out a detailed analysis of polymorphic CAG/GGN alleles and also found that min. 21 CAG and min. 24 GGN repeats simultaneously significantly increase the relative risk of sterility (severe hypospermatogenesis) by a factor of 1.6 (89). A smaller survey also showed worse sperm motility in case of longer CAG and GGN repeats (103). On the other hand, Brokken et al. (114) examined fertile Caucasian men (*N* = 557), and found that those with shorter than 23 GGN repeats (*N* = 44) had higher concentration of inhibin B, higher levels of progressive sperm and of correct morphology, and a higher fraction of Fas-positive sperm. Men with min. 24 GGN (*N* = 153) or min. 25 CAG (*N* = 118) both had higher estradiol levels, while those with 23 or fewer CAG had higher sperm DNA fragmentation (114).

GGN/polyG polymorphisms of AR may also correlate with certain measures of cognitive performance and risks of neurological conditions. For example, in a Chinese cohort of healthy individuals, a significant association was detected between polyG length and verbal memory of women (120). While in a Swedish cohort, a significant relationship was found between AR repeat polymorphisms and neuroticism or somatic anxiety, with an overrepresentation of people having short polyQ and long polyG repeat regions simultaneously (110).

### The non-polymorphic polyproline region of human androgen receptor

Androgen receptor-NTD also contains a polyproline stretch (polyP at aa. 374-381) of eight amino acids, consecutively, which is conserved from human to rodents (Supplementary File 1). Substitution of prolines in polyP is relatively rare, however leucines, alanines and histidines do occur in more than one species, while serine is only observed in spotted hyena and threonine in the last position of polyP in rodents.

Structurally, polyP sequences are known to fold into polyproline helices, and AlphaFold (128) does model this specific region of AR as a polyproline helix as seen in the AlphaFold Protein Structure Database (129). Functionally, proline-rich regions are known to be recognized by SH3 domains. Migliaccio et al. (130) showed that the SH3 domain of Src can actually bind polyP of AR, while interaction between AR and Src lacking the SH3 was barely detectable, and binding between AR lacking the polyP region and Src was undetectable. Deleting the C-terminus of polyP and its flanking region only exhibited very weak activation of Src (131). Furthermore, titration with this synthesized peptide (Ac-PPPHPHARIK-NH2) could also inhibit the AR-Src interaction (131). Moreover, another SH3-containing protein SH3YL was also proposed as a binding partner of AR’s polyP. Blessing and coworkers used a phage display to confirm the interaction with SH3YL1 and also concluded that the disruption of AR-NTD’s polyP reduced the hormone-dependent proliferation and migration of PCa cells (132).

It is also noteworthy that huntingtin, the protein involved in Huntington’s disease, also has mono-amino acid repeats of both polyQ and polyP. For this protein, it was demonstrated that the polyP region chaperones the polyQ region, and without polyP the polyQ repeat is more prone to aggregation (45, 133–136). It definitely would be interesting to study if the short polyP region of AR also chaperones its polyQ region, affecting its aggregation propensity.

The important role of the polyP region of AR is also highlighted by its sensitivity to mutations. The P380R substitution is cataloged in AIS (137), causing partial androgen insensitivity with ambiguous genitalia and sexual underdevelopment (138). Using luciferase reporter assays, it was demonstrated that the P380R substitution significantly reduces the hormone-induced transactivation of AR to ∼20% of the wild-type, thereby highlighting the mechanistic details of how this mutation causes AIS (139).

## Evolution and phylogenetics of the polymorphic repeat regions

Type 1 NHRs are a major and well-studied group of NHRs that bind bipartite hormone elements in homodimeric form (140). They evolved in a way that AR, PR, GR, and MR diverged from ER alpha and beta (141–143). Of the four type 1 steroid receptors, AR seems to be the most distant from ER-alpha (Id = 15.6%) (141, 143). The DBDs and LBDs of nucleic hormone receptors are well-conserved, most of their divergence arises from the intrinsically disordered NTDs that differ both in length and sequence (Figure 1, Supplementary File 1), which is not surprising for regions with structural disorder (144–149).

Across mammalian species, AR’s DBD and LBD are fully conserved with only a single amino acid (glutamate) insertion in the sheep DBD, two mismatches in the DBD and one mismatch in the LBD of spotted hyena (Supplementary File 1). These positions either correspond to the C-terminal end of the DBD fold and probably enable flexible motion of the domain with respect to the hinge region and LBD or represent part of the DBD fold but are not in the close proximity of DNA (hyena’s S614P using human AR numbering/S596P using rat AR numbering). While in case of the hyena LBD, the mismatch E838D (human AR numbering) is also distant from the steroid hormone binding pocket. However, E838 is located in a druggable cleft of AR-LBD, for example flufenamic acid and tiratricol interacts with it (150).

Going further in evolution toward vertebrates, there is a high degree of conservation of DBD, which can be explained by its function to bind to conserved DNA recognition elements (151). Mutation on the ARE site and/or the DBD could result in a disrupted signaling cascade (152). Therefore, the DBD remained practically unchanged for at least 500 million years (152, 153). The mutations in humans compared to fishes in the DBD resulted in AR being able to bind to other hormone response elements as well as increasing transcriptional activity (154). LBD is less conserved than DBD in a longer evolutionary context, still it is highly conserved and diverged significantly less during evolution than the LBD of other SRs (142).

**FIGURE 2:**
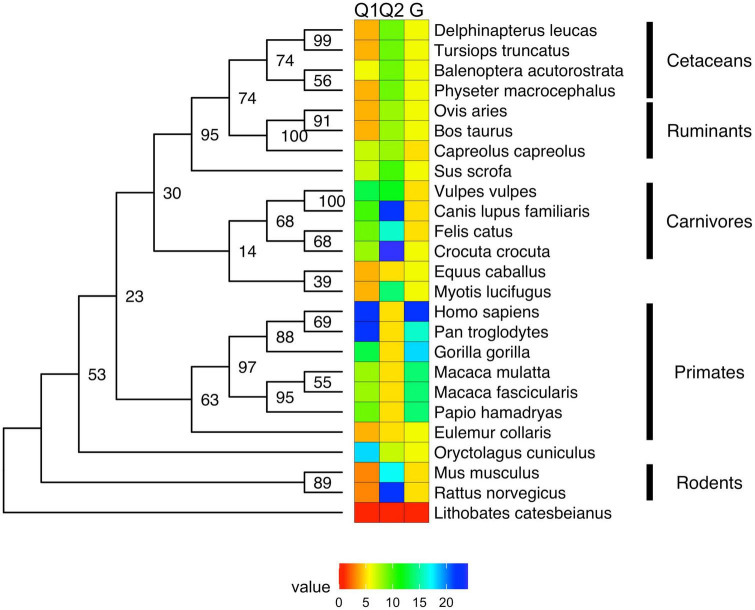
Maximum likelihood (ML) phylogenetic tree of androgen receptor with heat map showing the length of the polyQ and polyG repeats. The ML phylogenetic tree (here shown as a cladogram) is displayed for the full-length UniProt sequences of AR (33). Sequences were aligned by MAFFT (159) and refined by RAxML (160, 161), and numeric values on the tree of the aligned AR sequences (159) indicate bootstrap percentages from 2000 iterations (160, 161) that can be regarded as a confidence score of local tree topology.

Androgen receptor-NTD conservation across mammalian species is also relatively high with the exception of the polymorphic polyQ1 and polyG regions, however, non-mammalian vertebrates have significantly lower conservation of the NTD (142, 155), and are completely devoid of polyQ and polyG regions (Figure 2). Within mammals, the most conserved parts of the NTD is the extreme N-terminal 35 residues preceding the polyQ1 stretch and region 231–255 (human AR numbering) harboring a putative CRM1 nuclear export signal (144, 156, 157). This region is also responsible for the interaction with Hsp70 (158) and has been reported to be amyloidogenic (60, 61).

**FIGURE 3:**
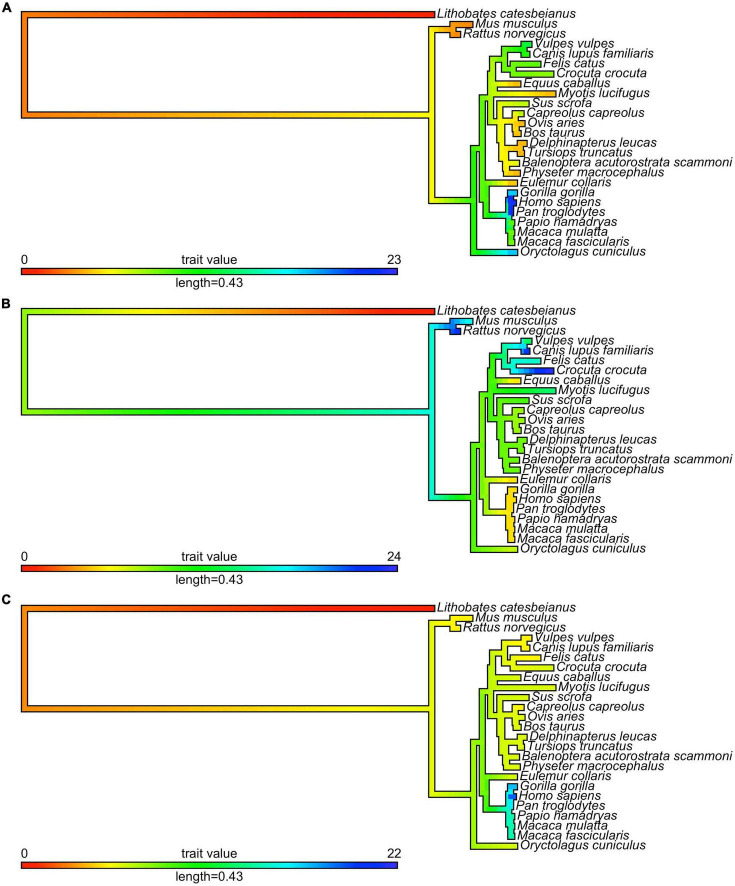
Evolution of mono-amino acid repeat lengths in androgen receptor. The same ML tree as on Figure 2 (here shown as a phylogram) was colored according to the reconstructed length of polyQ1 (panel **A**), polyQ2 (panel **B**), and polyG (panel **C**) repeats using the phytools R package (162) and its fastAnc and conMap commands.

In this article, we present a multiple sequence alignment (Supplementary File 1) and corresponding phylogenetic tree showing the evolution of polymorphic regions in mammalian species (Figures 2, 3). The overall topology of the maximum likelihood (ML) protein tree (Figure 2) reveals some divergence from nuclear phylogenies of mammals (163, 164). Rodents (*Mus musculus*, *Rattus norvegicus*) and rabbits (*Oryctolagus cuniculus*) occupy a basal position, decisively distant from the primates’ clade, to which it is closely related in the nuclear phylogenies. Another notable exception is the bat’s AR, which appears to be closest to horses, and is positioned in a very nested position within the phylogeny. It is also worth mentioning that bat (*Myotis lucifugus*) appears to have the longest branch length among all the species considered, suggesting that its AR amino acid sequence has diverged the most compared to all the other orthologs considered, based on its position on Figure 3.

With this considered, one can appreciate that a significant part of these small changes among mammalian ARs occur around polymorphic regions. The polymorphic polyglutamine repeat (polyQ1) features in higher primates, being shorter or interrupted with other residues or even absent from mammals to frog and fish, which is also the case for other polyQ-containing and neurodegenerative disease related proteins (42). Two early studies tried to shed light on the timeline of divergence of polymorphic regions in mammals (165, 166). Despite the importance of these pioneering works, it cannot be ignored that the sample size of primates was very small, and apes were compared to rats as mammalian controls, which leads to biased conclusions as the rodents have shown to be outliers by the phylogenetic tree.

The conservation of the 22/23-residue-long polyQ1 region is quite poor, i.e., polyQ1 stretches of at least 14 glutamines can only be found in human and chimpanzee AR-NTD (considering the most common allele) (Figure 2). Interestingly, only the sequence of the human and of a few apes (chimpanzee, gorilla) have 3–4 leucines as N-terminal flanking “gatekeeper residues” of the polyQ1 region, the other mammals with shorter CAG repeats have a single leucine as flank, suggesting that the length of the leucine-stretch correlates with the polyQ1 length in evolution. After apes, carnivores have the next longest polyQ1 regions (8–10), followed by Old World monkeys (8–9) and then New World monkeys (4) (Figure 2). Pigs have longer polyQ1 region (7) than New World monkeys, but odd-toed ungulates, ruminants, cetaceans and bats generally have 4-residue-long polyQ1. The shortest polyQ1 region is in rodents with a length of 2 (Figure 2). The CDK phosphorylation site XX([ST])P[RK] immediately adjacent to the polyQ1 (ETSPR) is also well-conserved in ARs with only little variation across mammalian species (Supplementary File 1). Following the phosphorylation site, most mammals have 4–8 more glutamines, with the exceptions of cats that have 11, and rodents that only have 2 glutamines and the rest of the glutamines are mutated to arginine or histidine. The outgroup species considered for the calibration of our trees (the frog species *Lithobates catesbeianus*) completely lacks the polyQ1 repeats (Figure 2), highlighting a trend of acquiring the polyQ1 region and increasing its length in mammals, particularly in the apes’ clade. This is clearly elucidated through ancestral reconstruction displayed on the tree in Figure 3A. The ancestral state is predicted to have polyQ1 length of 2, which disappeared in amphibians after divergence. Although in rodents the ancestral length is maintained, an overall increase of polyQ1 is observable in the rest of the species, with an average of ∼12 repeats. While carnivores retain this state, a few reversals (i.e., subsequent decrease in polyQ length) are observed: in the case of bat (*M. lucifugus*), horse (*Equus caballus*) and the Artiodactylian clade (Cetaceans and Ruminants), which show 4–10 polyQ1 repeats. In the primate clade, a range of repeat lengths is observed, from the 2 of the basal lemur (*Eulemur fulvus collaris*) to the 23 repeats in *Homo sapiens*, the latter representing the highest value observed in this set of sequences (Figure 3A). An interesting case is the one of rabbit (*O. cuniculus*), which shows a long polyQ1 repeat (∼15) despite its early divergence in the phylogeny (Figure 2).

Polyglutamine repeat 2 is located C-terminally ∼115 amino acids away from polyQ1. An opposite trend is observed in the evolution of these repeats (Figures 3A,B). The most ancient mammalian ancestor reconstructed here is predicted to possess a polyQ2 repeat of ∼12 residues (Figure 3B). Bullfrog (*L. catesbeianus*) lacks the polyQ2 repeat, while the ancestral state is maintained throughout most of the mammalian evolution (Figure 3B). In contrast to polyQ1, basal clades like rodents show the longest polyQ2 repeats (22–24) and primates bear the shortest ones (∼5 glutamines), and the two polyglutamine stretches are slightly (inversely) correlated (Spearman’s *R* = −0.16). Interestingly, carnivores and rats have the longest (min. 20 residues) polyQ2, but often interrupted by arginines or histidines (Supplementary File 1).

The polyG region of AR is ∼86 amino acids N-terminally from AR-DBD, and it is longest in humans (∼23 residues), followed by apes (17–22 residues), Old World monkeys (15 residues), New World monkeys (8–14 residues) and other non-primate species (<10 residues) in order (Figure 2). Its evolution shows a similar trend to polyQ1, with an ancestral condition of ∼4 repeats, no polyG in bullfrog (*L. catesbeianus*), a basal state of 5–10 repeats for most of the mammalian species (Figure 3C). The exception is the primate order, which shows a rapid increase in the number of glycine repeats (22 in human). It is of note again, that glycines of polyG are also sometimes mutated to other small amino acids (serines, threonines, and alanines). In most mammals, the polyG region is flanked N-terminally by a cysteine and C-terminally by a glutamate (Supplementary File 1). In mouse and rat, the cysteine is missing or substituted by glycine (5 uninterrupted glycines in a row); moreover, the glutamate is replaced by aspartate, while the second half of the polyG is mutated to 451-SSSPS-455 (rat AR numbering). This highlights how far most mammalian AR evolved from those of rodents. Interestingly, we also confirmed the significant correlation between polyG and polyQ1 length (Spearman’s *R* = 0.47), furthermore significant inverse correlation between polyG and polyQ2 length (Spearman’s *R* = −0.54) across mammalian species.

In summary, mono-amino acid repeats do not occur in non-mammalian species, not even the non-polymorphic polyP despite its high conservation of sequence (max. 2 substitutions) and length (eight amino acids) across mammals (Figure 2).

Repeat polymorphisms across individuals within a species was not found to be unique to the human AR (Table 1), polyQ1 and polyG length also varies in other primates. In chimpanzees (*N* = 89) the polyG ranges between 14 and 22 repeats with 17–19 being the most common, while in bonobos (*N* = 54) only alleles with 18 and 19 repeats were found with 87 and 13% frequency (167, 168). Two independent studies have concluded that polyG and polyQ1 lengths are inversely correlated in chimpanzees (167, 169). In common squirrel monkeys (*N* = 10) polyG ranges between 21 and 24, with 21 being the most frequent allele, while polyQ length stays invariantly 4 + 5 (polyQ1 + polyQ2) (169). In tufted capuchin monkeys (*N* = 47) polyG varies between 11 and 14, and similarly the fewest repeats (11) being the most frequent allele; by contrast, the length of polyQ regions does not vary much and stays 5 + 5 or less frequently 5 + 4 (polyQ1 + polyQ2) (169). Surprisingly, both squirrel monkeys and tamarins were found to have a significantly higher number of GGA glycine codons (29 and 42%) in polyG in comparison to Old World monkeys and apes that are devoid of this codon in their polyG region (169). The polymorphic polyG and polyQ1 length did not (inversely) correlate in New World monkeys the same way as it did in chimpanzees (169).

Polyglutamine repeat polymorphism in AR outside primates has also been discovered in a few carnivores. In the polyQ1 of red foxes (*N* = 181), most frequent CAG allele had 10 repeats, both in males and females (65.85 and 57.39%, respectively), followed by 10T (24.39 and 31.25%)–meaning one CAG was mutated to CAT–, then 13 repeats (7.32 and 9.09%) and finally 12 repeats (2.44 and 2.27%, respectively) in order (170). Interestingly, uninterrupted CAG10 was more common in aggressive female foxes than in curious females, while CAT/His interrupted CAG10 was less common in aggressive female foxes than in curious females (170). In the polyQ1 and polyQ2 of healthy dogs (*N* = 172), three polyQ1 alleles (with 10, 11, and 12 CAG) and three polyQ2 alleles (with 11, 12, and 13 CAG) were discovered with 11 being the most common in both of them (48.8 and 75.6%, respectively) (171). Interestingly, in the Doberman dog breed (*N* = 31) polyQ1 with 10 CAG was way more common (67.7%) than 11 CAG (32.3%), and 12 CAG was not represented at all despite the 18.6% expected occurrence (171). Doberman was the only guard dog breed in this present study, which raises the question whether the shorter polyQ1 region contributed to making this dog breed fearless, zealous, and fierce. Similarly to men, genotyped dogs with canine PCa (*N* = 31) had a tendency for shorter polyQ1 length with 10 or 11 CAG repeats (54.8 and 45.2%, respectively), while none of the dogs with PCa had 12 CAG in polyQ1 (171). Ochiai et al. (172) tested recombinant canine AR with polyQ1 of 9–12 glutamines in PC3 cells and found that constructs with shorter polyQ1 had significantly higher activities than those with longer polyQ1 (luciferase assays). As most male dogs are castrated when young, PCa cases not responding to hormonal androgen ablation and AR antagonists are very common, hence prognosis is as poor as in human CRPC (172), and radio- or chemotherapy and radical prostatectomy remain as last resorts.

## Controversies

Although the relationship between fewer CAG repeats in polyQ1 and increased risk of PCa is seemingly established, it is still debated (173). For example, in a large multi-ethnic cohort of more than 4,000 men, Freedman et al. (102) did not find a significant relationship between CAG repeat length and the risk of PCa, similarly to smaller studies on the Caucasian population (100, 101, 121) (Table 1A). Controversies also exist with regards to the GGC repeats of the polyG region: for instance, the GGC repeats alone did not exhibit correlation with PCa risk in Scottish men (101), in the African American population (112), in Nigerian men (113), and in the Turkish population (174) (Table 1B).

Another point of debate is the negative correlation between the polymorphic CAG and GGC repeats, as various studies confirmed it (98, 112, 115), while others could not provide conclusive evidence (99, 109). The correlation was also demonstrated in chimpanzees (167, 169), however no correlation was observed in New World monkeys (169).

Yet another disputed area is the transcription activity of AR, which is generally thought to be inversely correlated with the length of both CAG repeats and GGN repeats. Nonetheless, some early studies were unable to detect these differences with varying CAG repeats (175, 176). Although in each of these studies, the template of the AR gene for further cellular experiments was isolated from two patients diagnosed with SBMA, the cell lines used in the experiments were neither prostate nor even human cells. Increased PSA levels are claimed to be reflective of overstimulated transcription activity of AR with shorter polyQ1/CAG repeats (109, 113, 177), however this correlation was not detected by others (117, 178). Interestingly, Bennett et al. (73) found almost three times higher PSA concentration in African-American than in Caucasian PCa patients, and in the same time median length of 20 vs. 22 CAG repeats, respectively.

Furthermore, more research is clearly needed to clarify whether CAG repeats encoding for polyQ1 can influence cognitive function. An interesting study assessed cognitive impairment (problems with thinking, communication, understanding and memory) by Mini-Mental State Examination (MMSE) in predominantly Caucasian elderly men, and found an association between longer CAG repeats and poorer performance (179). However, Kovacs et al. (120) found no relationship between CAG repeats and memory function in the Chinese population. On the other hand, the latter study surprisingly reported that GGN repeat length may affect verbal memory, which was not tested by the MMSE study on elderly female subjects. Notwithstanding, the lack of consistency in results may stem from the difference in cohort subjects with regards to age, ethnicity, and gender.

## Current research gaps

In this review, we summarized efforts in determining the risk of length variations in the polymorphic regions of AR to certain types of cancer. These studies have mostly focused on a specific population in a single country, aside from a few exceptions, including the studies by Ackerman et al. (68) and Kittles et al. (74). As elucidated in the previous sections, these may explain controversies and ongoing debates about associations detected by some studies but not by others. In the coming years, it would be important to clarify the source of these differences by larger and more diverse collaborative or consortium-led surveys to resolve which exact polymorphism (and combination of alleles) have what effect. The advantage would be a great deal of control over the methodology (no arbitrary grouping, no limitation due to small sample size, same comparisons, tests, and metrics), the clinical parameters and biomarkers measured could be harmonized, and the diverse cohort could reveal new differences in ethnicity, age and gender. Furthermore, it is important to emphasize that anonymized raw data was very rarely shared along with the publication (link to data, Supplementary material), which would be largely beneficial for smooth accessibility, reproducibility, and reusability for meta-data analyses.

Studies exploring the polymorphic nature of polyQ1 and polyG repeats in certain subpopulations, and the clinical associations thereof are dominant in the literature, nonetheless efforts should also focus on the exact mechanisms how these regions function. It is still not totally clear, how longer GGC repeats result in lower protein abundance, and whether shorter than average GGC repeat length could result in higher intracellular concentration. PolyG length certainly varies across species from short to long (see Section “Evolution and phylogenetics of the polymorphic repeat regions”), which tempts us to wonder if AR abundance is again higher in those animals in comparison to humans. Moreover, what is the interplay between polyG/GGC repeats and polyQ1/CAG repeats that makes them correlate throughout evolution? Does the polyG/GGC stretch affect the structure and function of AR on the protein level, or it only regulates the translation efficiency on transcript level? For example, the polyG region is adjacent to the binding segments of the ralaniten-like drug candidates (11, 63, 180). It would be interesting to know if polyG length has an effect on the binding of the compound. Also, polyQ length was shown to readily modulate the NTD-LBD interaction (26), but more research should be dedicated to explore its effect on binding other macromolecular partners, as well (21, 24, 181).

A key missing area to further explore, is the modulatory role of polyQ1 and polyG and the effect of their length, respectively, as well as in certain combinations on biomolecular condensation, i.e., the formation of nuclear foci, by AR. It has been known for a long time that many TFs have a non-homogeneous distribution in the nucleus and form foci (or also termed nuclear puncta) at the DNA target site (182–186). Liquid-liquid phase separation (LLPS), a recently emerged phenomenon, provides a mechanistic explanation to the formation of these biological condensates, which has been detailed in recent reviews (187–189). LLPS is a thermodynamically driven reversible phenomenon present from bacteria to humans, and also in plants, reported to be involved in many biological processes and diseases (190–193). Upon LLPS, two separate phases of substantially different concentration and viscosity form, giving rise to a low concentration dilute phase and a high concentration condensed phase (194). Many TFs–including nuclear receptors GR, ER and AR–have been indicated to undergo LLPS (195–197). Moreover, other important transcriptional machinery proteins (e.g., MED1) were demonstrated to drive condensate formation, while others such as RNA polymerase II were shown to be recruited to the condensates–in both cases via their low-complexity intrinsically disordered regions (IDRs)–suggesting that LLPS have an important role in transcriptional regulations (196, 198). Due to their multivalency, IDRs are often considered to be potential drivers of condensate formation (199). In case of AR an early access preprint manuscript reported that only full-length AR can undergo LLPS upon ligand binding, and ARv7, which contains the unstructured low-complexity NTD but lacks the globular LBD, did not show condensate formation (200). They also showed that upon disruption of condensate formation the transcription activity was inhibited as well, suggesting that it has a crucial role in the regulation of AR activity. Another study verified that ARv7 and AF1 (aa. 144-488) were unable to undergo LLPS alone or in the presence of RNA mimic polyU (201). However, the AR-DBD was identified as a minimal region capable of driving LLPS in the presence of polyU (201). Another recent study showed that the length of the polyQ affects nuclear localization and hence the transcriptional activity of AR (202). This suggests that despite AR-NTD being insufficient for driving LLPS alone, it still has a regulatory role, probably by determining solubility via the length of the polyQ1 and recruiting co-factors that can alter the LLPS propensity. However, this research direction is still poorly understood, although there is increasing attention in the cell biology field to explore this new modality for regulation of certain molecular functions. For example, AR can constitute part of enhanceosomes (200, 202–204), hence overactivation of transcription in cancer should also be studied in the contexts of liquid-like phase separated condensate-state. The importance of LLPS raises the mechanistic question of the role of different domains (driver, regulatory or passive region), and polymorphic and splice variants, of AR in biomolecular condensate formation (transcriptional condensates, enhanceosomes) in late stages of PCa.

Another interesting, yet undiscovered area is related to long non-coding RNAs (lncRNAs). These RNAs comprise a huge part of the human transcriptome (205) and are subject of intense research due to their indication in many important cellular regulatory processes and cancer implications (206). AR has been reported to interact with several PCa-related lncRNAs, such as *HOTAIR*, *PCAT1*, *HOXA11-AS-203*, *SOCS2-AS1*, *LBCS*, *GAS5* with a poorly understood mechanism (207, 208). In advanced PCa cell lines many of these lncRNAs are either upregulated or downregulated, further strengthening the relevance of the need to understand the molecular mechanism of these interactions. PCa-related lncRNAs have been summarized in a recent review in detail by Yang et al. (207). It is of high relevance, that the interaction between *SLCNR1*, a melanoma-related lncRNAs and AR was reported recently (209, 210). The authors identified a pyridine-rich motif that they proposed as a canonical AR-NTD binding motif, as it exists in other AR interacting lncRNAs, such as *HOTAIR* and *HOXA11-AS-203* (211). In a follow up study, the same group successfully targeted the binding motif by oligonucleotides sterically blocking the interaction and thereby attenuating *SLNCR1*-mediated melanoma invasion (211). The NTD used by the authors in the studies contained only the most frequent polyQ1 and polyG length. It would be interesting to compare the binding of NTD with different polymorphic variants to lncRNAs to shed light on the possible direct or allosteric effect of mono-amino acid repeat length.

RNAs often facilitate LLPS (212), which has already been reported regarding the DBD of AR (201). Furthermore, many lncRNAs form ribonucleoprotein condensates, which are important in transcription (213, 214). Therefore, it would be important to study the effect of lncRNAs on AR’s LLPS behavior with different lengths of the polymorphic regions in pathophysiology, as it could shed light on future therapeutic windows to target these interactions.

Hopefully, addressing these research gaps will enable potential breakthroughs in understanding these polymorphisms and their cross-talk, with implications in diagnostics of patients with AR alleles representing moderate to high risk to certain diseases and in developing therapeutics that are not affected by these polymorphisms or therapeutics that counterbalance the effect of overly long or short alleles.

## Future directions and potential breakthroughs in the field

Given the pace with which molecular and cell biology, genetics, diagnostics, and drug discovery develop, one can foresee a number of potential breakthroughs in the field focusing on better understanding and modulating of AR. It would be crucial to understand the molecular mechanism of the LLPS behavior of AR with regards to its activity. Further systematic *in vitro* and *in vivo* studies are required to elucidate the contribution of the different domains as well as the two polymorphic regions to the condensate formation. Including co-factors and other crucial partners to these future studies could enable better understanding of the transition from physiological to pathological states, and explaining some of the controversies around these regions. Within NTD, elucidating the mechanism by which polyQ1 and polyG affects the functional repertoire of AR, would also enable the therapeutic targeting of these protein segments. Effect of polyQ1 length of AR on PCa and neurodegenerative disease like SBMA has been confirmed but there is a need for validation on the effect of polyG and polyP and their interplay before targeting. There are different possibilities to target repeat associated diseases for AR at DNA, RNA and protein level.

On RNA level one way of targeting these polymorphic regions is by antisense oligonucleotides (ASOs) and stabilized miRNA analogs, which inhibit the translation of mRNA, this represents a fast-developing modality of drug design for repeat-associated diseases like Huntington’s disease (215–217), myotonic dystrophy type-1 (218–220) and amyotrophic lateral sclerosis (221–223). ASO stability and delivery have been ongoing problems, but now there is a growing number of new technologies for delivery, like liposomes, to mitigate these difficulties, which make them very attractive for targeting repeat-associated diseases (224).

At the DNA level, another approach could be a CRISPR/Cas9-based therapy for genetic engineering to restore the wild-type repeat number of the polymorphic regions, a method that is already developed for other repeat-associated diseases like Huntington’s disease (225–227), Duchenne muscular dystrophy (228), myotonic dystrophy type-1 (229, 230), spinocerebellar ataxia type-3 (231), Friedreich’s ataxia (231, 232) and amyotrophic lateral sclerosis (233). Off-target effects of this technology has been initially a challenge, but there are intensive efforts on reducing it, e.g., by dual CRISPR/Cas9 technology (234).

Inhibiting intramolecular or intermolecular interactions of AR-NTD is yet another way of interfering with its pathogenic malfunctioning. However, this is particularly challenging due to the intrinsically disordered nature of the NTD. IDRs have been considered to be undruggable for a long while, although new success stories of upcoming molecules targeting IDRs have mostly dissolved this dogma (235–237), including the development of ralaniten and its further optimized versions (11, 63, 180). As a subcategory of small molecule targeting, induced degradation of AR, especially its pathological isoforms and alleles, by proteolysis targeting chimeric compounds (PROTACs), molecular glues and autophagosome-tethering compounds is also expected to lead to potential breakthroughs (238–242). This strategy enables to lower the intracellular concentration of AR, therefore downregulating downstream transcriptional signaling.

Recent advances in understanding phase separation of AR provide opportunities to modulate condensates, thus targeting enhanceosomes and transcriptional condensates of AR variants may hold the future for drug discovery (200, 203, 204, 243–245). Currently, condensate modulators are being conceived to inhibit LLPS, re-solubilize the condensates or dissolve the aggregates formed from condensate foci, or on the contrary to harden condensate for inactivation (246–250).

## Conclusion

Mono-amino acid repeats are present in many organisms including animals, plants, and fungi. For example, polyQ regions with increasing length affect solubility, stability, and abundance of proteins. Changes in hydrophobicity and secondary structure could result in oligomer formation, which potentially leads to condensation and aggregation.

Polyglutamine repeat regions are also located in AR-NTD with flanking regions exerting inhibitory effects on aggregation for both polyQ1 and polyQ2. PolyQ1 flanking region contains four leucines, while polyQ2 contains 2 leucines that act as aggregation gatekeepers. Mutation in leucine and/or polymorphism in polyQ1 can induce structural changes, which can result in different diseases. For example, shorter polyQ1 is associated with increased activity of AR, which can cause PCa and rheumatoid arthritis (87, 251). A longer length of polyQ1 results in aggregation and is associated with the neurological disease SBMA (252). PolyQ1 polymorphism and its effect on protein aggregation has been studied at the molecular level by different groups. It was shown that the N-terminus of AR can even form amyloids *in vitro*, and *in cellulo* aggregates in SBMA (61, 251).

Recently, it was reported that AR forms condensates in the nucleus, and elevated nuclear localization was observed despite decreased transcriptional activity with increasing length of polyQ1 (202). This work is very preliminary, and further studies will be needed to elaborate whether increase in polyQ1 length results in phase separation and/or aggregation. It would also be important to explore the effect of different lengths of polyQ1 on the dynamic liquid-like properties of these condensates, and how that affects the recruitment of different binding partners and resulting downstream signaling. Moreover, it would be also worth studying whether the length of the polyG has any effect on condensate formation.

It has been shown that length of polyG is associated with a decrease in its own translation and consequently transcriptional activity. Polymorphism in polyG length has been proposed to be associated with diseases including prostate and ECa, AIS, and neurological diseases. PolyG polymorphism has also been studied in relation to co-occurrence with polyQ1 repeats in pathology, and polyG ≤ 14 and polyQ1 ≤ 17 were found to be associated with PCa in the Caucasian population. Risk of being sterile increases for men with min. 21 CAG and min. 24 GGN repeats.

Even though the length polymorphism of polyG in AR has been studied at population level very intensively–although with controversial results –, there has not been a lot of work performed at the molecular level. Shorter polyG negatively correlated with PSA staining, especially in the more severe type of PCa with higher Gleason scores, but it would be important to investigate how polyG interplays with polyQ1, and how changes in lengths affect the phase separation and protein aggregation properties and consequently the resulting phenotype. Answering these questions will help understand the mechanism by which these polymorphic repeats function from shorter to longer alleles of the population.

It is little discussed that AR-NTD also contains an 8-amino acid-long stretch of polyP. It has been shown that disruption of the polyP–SH3YL1 interaction results in reduced hormone-dependent proliferation. It is of note that in cases of other proteins, e.g., huntingtin, it was shown that the polyP segment can chaperone the adjacent polyQ region. However, further research is needed to test if such chaperoning also applies to AR.

Overall, there are several knowledge gaps that hinder the understanding of these repeats in AR, and also of their crosstalk, i.e., how this interplay at molecular level brings changes at population level. These need to be explored further and studying this will provide opportunities to find ways of targeting diseases and potentially also to transfer this knowledge to other repeat-rich proteins with similar build-up like AIB1/NCOA3, SK3 and huntingtin.

Targeting of AR-NTD is especially challenging due to the intrinsically disordered nature of the region and the limited coverage of NTD with detailed structural characterization. So far, only ralaniten and its further developed successors has been shown to bind AR-NTD (covalently or with sufficient affinity) with properties compatible with drug development. One can expect to see more studies directly addressing the influence of the adjacent polyG region with length polymorphism, or the effect of the partially interacting polyQ1 region on the drug binding properties of transactivation unit AR-Tau5 in the coming years. Alternative to protein-protein/DNA interaction inhibitors, targeted protein degradation inducing compounds offer a complementary approach to interfere with the overactive signaling or aggregating oligomers of a protein. To the best of our knowledge PROTACs against AR are all based on drugs interacting with LBD, however one could also envision degraders developed from ralaniten-like compounds. Moreover, therapeutic targeting of AR can also concentrate on the DNA and mRNA level by CRISPR/Cas9-based technologies and ASOs, which methods have a relatively lower entry barrier for drug development but well-known challenges in delivery and stability. However, as AR phase separates in the nucleus to form transcriptional condensates (e.g., overactive enhanceosomes in PCa) it might be an important property for drugs to be able to partition into these condensates to exert their effects. Alternatively, condensate modulators can also be applied to hinder LLPS formation or dissolve condensates and thereby inhibit the constitutive transcriptional activation. As the number of investigational condensate modulators are rapidly growing, it is not far-fetched to expect these compounds to come of age in the near future.

## Author contributions

TL, AM, JA, and GR: conceptualization, investigation, and writing – original draft. GR and TL: visualization. TL: project administration. TL and PT: supervision. TL, AM, JA, GR, and PT: writing – review and editing. PT: funding acquisition. All authors contributed to the article and approved the submitted version.

## Supplementary material

Supplementary File 1 Multiple sequence alignment of mammalian androgen receptor protein sequences and androgen receptor of American bullfrog (*Lithobates catesbeianus*) as an outgroup. All sequences are publicly available from UniProt with the accession code preceding the sequences.

## Abbreviations

AIS: androgen insensitivity syndrome
AR: androgen receptor
ARE: androgen response element
ARv7: androgen receptor splice variant 7
ASO: antisense oligonucleotide
BPH: benign prostate hyperplasia
BRCa: breast cancer
CRPC: castration-resistant prostate cancer
DBD: DNA-binding domain
ECa: endometrial cancer
ER: estrogen receptor
GR: glucocorticoid receptor
HSP: heat shock protein
IDP: intrinsically disordered protein
IDR: intrinsically disordered region
LBD: ligand-binding domain
LLPS: liquid-liquid phase separation
lncRNA: long non-coding RNA
ML: maximum likelihood
MR: mineralocorticoid receptor
NHR: nuclear hormone receptor
NMR: nuclear magnetic resonance
NTD: N-terminal domain
PCa: prostate cancer
polyG: polyglycine repeat
polyP: polyproline repeat
polyQ: polyglutamine repeat
PR: progesterone receptor
PROTAC: proteolysis targeting chimeric compounds
PSA: prostate specific antigen
SBMA: spinal-bulbar muscular atrophy
SH3: Src Homology 3 domain
TF: transcription factor.
