# Supplementary material for: The evolution and polymorphism of mono-amino acid repeats in androgen receptor and their regulatory role in health and disease
Source: Front Med (Lausanne). 2022 Oct 20;9:1019803. doi: 10.3389/fmed.2022.1019803 (PMC9642029; doi:10.3389/fmed.2022.1019803)
Supplement: Supplementary File 1 — Multiple sequence alignment of mammalian androgen receptor protein sequences and androgen receptor of American bullfrog (Lithobates catesbeianus) as an outgroup. All sequences are publicly available from UniProt with the accession code preceding the sequences. [file Data_Sheet_1.PDF]

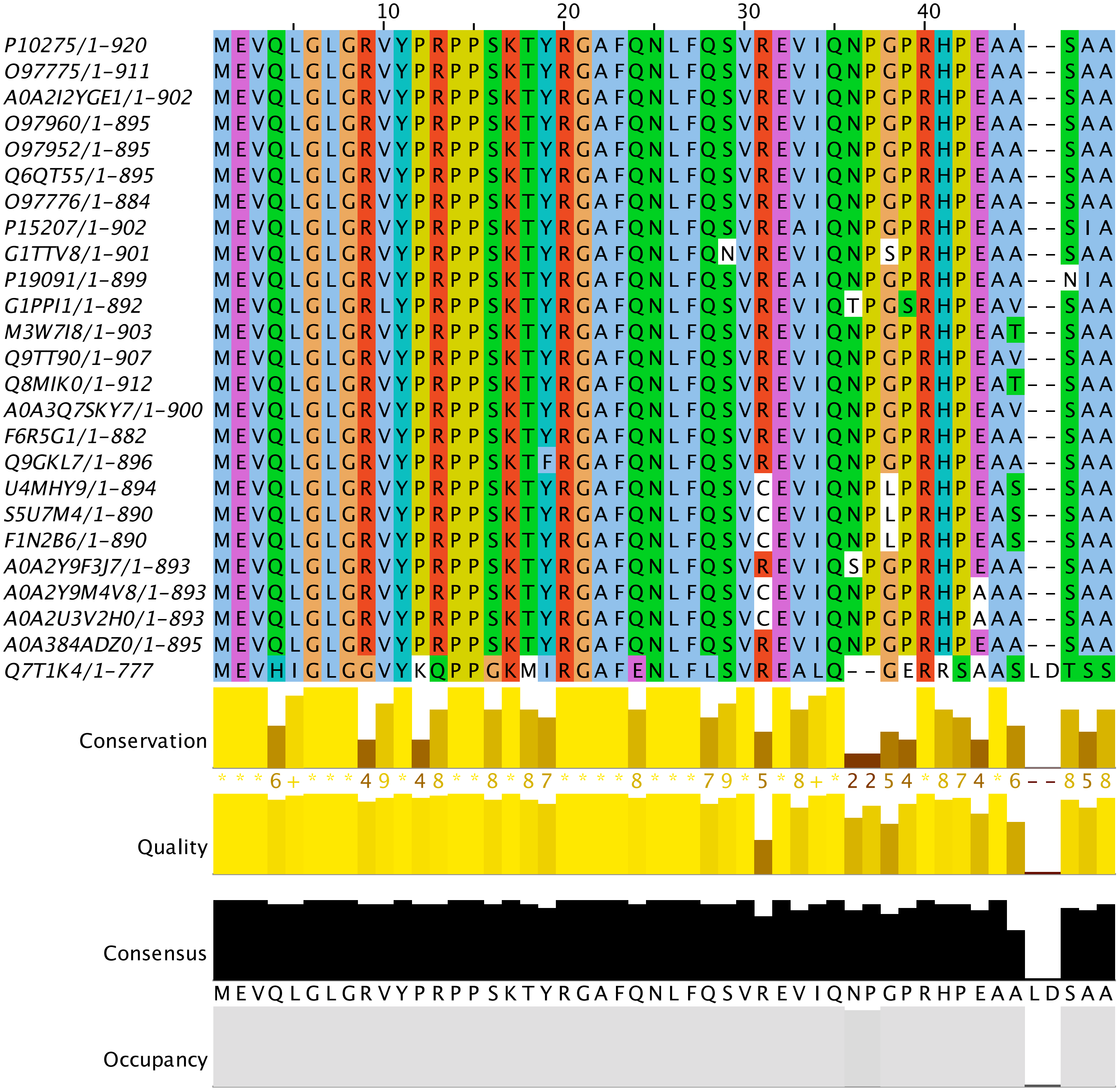

10 20 30 40
P10275/1-920 MEVQLGLGRVYPRPPSKTYRGAFQNLFQSVREVIQNPGPRHPEAA--SAA
O97775/1-911 MEVQLGLGRVYPRPPSKTYRGAFQNLFQSVREVIQNPGPRHPEAA--SAA
A0A2I2YGE1/1-902 MEVQLGLGRVYPRPPSKTYRGAFQNLFQSVREVIQNPGPRHPEAA--SAA
O97960/1-895 MEVQLGLGRVYPRPPSKTYRGAFQNLFQSVREVIQNPGPRHPEAA--SAA
O97952/1-895 MEVQLGLGRVYPRPPSKTYRGAFQNLFQSVREVIQNPGPRHPEAA--SAA
Q6QT55/1-895 MEVQLGLGRVYPRPPSKTYRGAFQNLFQSVREVIQNPGPRHPEAA--SAA
O97776/1-884 MEVQLGLGRVYPRPPSKTYRGAFQNLFQSVREVIQNPGPRHPEAA--SAA
P15207/1-902 MEVQLGLGRVYPRPPSKTYRGAFQNLFQSVREAIQNPGPRHPEAA--SIA
G1TTV8/1-901 MEVQLGLGRVYPRPPSKTYRGAFQNLFQNVREVIQNPSPRHPEAA--SAA
P19091/1-899 MEVQLGLGRVYPRPPSKTYRGAFQNLFQSVREAIQNPGPRHPEAA--NIA
G1PPI1/1-892 MEVQLGLGRLYPRPPSKTYRGAFQNLFQSVREVIQTPGSRHPEAV--SAA
M3W7I8/1-903 MEVQLGLGRVYPRPPSKTYRGAFQNLFQSVREVIQNPGPRHPEAT--SAA
Q9TT90/1-907 MEVQLGLGRVYPRPPSKTYRGAFQNLFQSVREVIQNPGPRHPEAV--SAA
Q8MIK0/1-912 MEVQLGLGRVYPRPPSKTYRGAFQNLFQSVREVIQNPGPRHPEAT--SAA
A0A3Q7SKY7/1-900 MEVQLGLGRVYPRPPSKTYRGAFQNLFQSVREVIQNPGPRHPEAV--SAA
F6R5G1/1-882 MEVQLGLGRVYPRPPSKTYRGAFQNLFQSVREVIQNPGPRHPEAA--SAA
Q9GKL7/1-896 MEVQLGLGRVYPRPPSKTFRGAFQNLFQSVREVIQNPGPRHPEAA--SAA
U4MHY9/1-894 MEVQLGLGRVYPRPPSKTYRGAFQNLFQSVCEVIQNPLPRHPEAS--SAA
S5U7M4/1-890 MEVQLGLGRVYPRPPSKTYRGAFQNLFQSVCEVIQNPLPRHPEAS--SAA
F1N2B6/1-890 MEVQLGLGRVYPRPPSKTYRGAFQNLFQSVCEVIQNPLPRHPEAS--SAA
A0A2Y9F3J7/1-893 MEVQLGLGRVYPRPPSKTYRGAFQNLFQSVREVIQSPGPRHPEAA--SAA
A0A2Y9M4V8/1-893 MEVQLGLGRVYPRPPSKTYRGAFQNLFQSVCEVIQNPGPRHPAAA--SAA
A0A2U3V2H0/1-893 MEVQLGLGRVYPRPPSKTYRGAFQNLFQSVCEVIQNPGPRHPAAA--SAA
A0A384ADZ0/1-895 MEVQLGLGRVYPRPPSKTYRGAFQNLFQSVREVIQNPGPRHPEAA--SAA
Q7T1K4/1-777 MEVHIGLGGVYKQPPGKMIRGAFENLFLSVREALQ--GERRSAASLDTSS
Conservation
* * * 6 + * * * 4 9 * 4 8 * * 8 * 8 7 * * * * 8 * * * 7 9 * 5 * 8 + * 2 2 5 4 * 8 7 4 * 6 - - 8 5 8
Quality
Consensus
MEVQLGLGRVYPRPPSKTYRGAFQNLFQSVREVIQNPGPRHPEAALDSAA
Occupancy

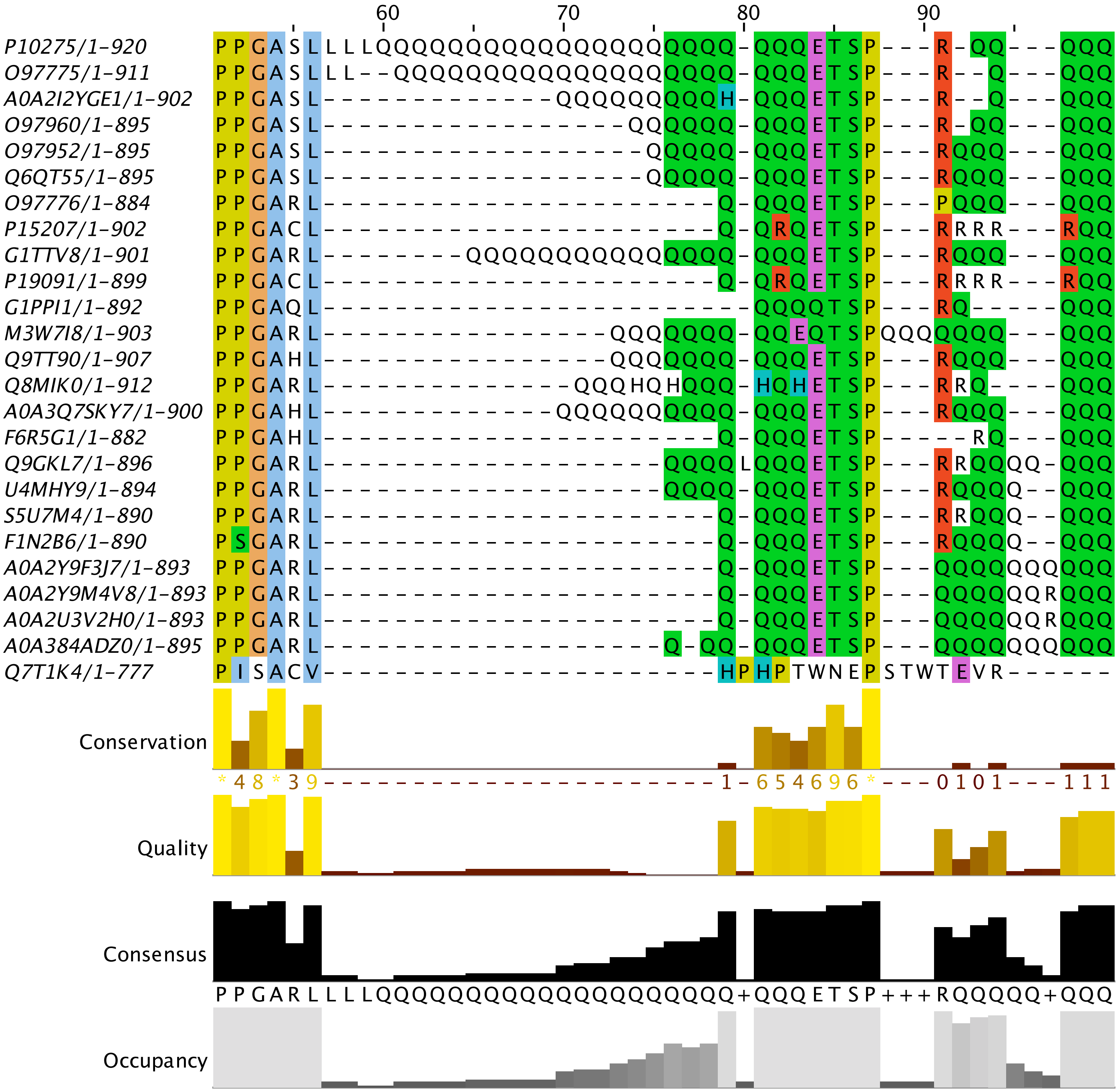
60 70 80 90
P10275/1-920 PPGASLLLLQQQQQQQQQQQQQQQQQQQQ-QQQETSP---R-QQ---QQQ
O97775/1-911 PPGASLLL--QQQQQQQQQQQQQQQQQQQ-QQQETSP---R--Q---QQQ
A0A2I2YGE1/1-902 PPGASL-------------QQQQQQQQQH-QQQETSP---R--Q---QQQ
O97960/1-895 PPGASL-----------------QQQQQQ-QQQETSP---R-QQ---QQQ
O97952/1-895 PPGASL------------------QQQQQ-QQQETSP---RQQQ---QQQ
Q6QT55/1-895 PPGASL------------------QQQQQ-QQQETSP---RQQQ---QQQ
O97776/1-884 PPGARL----------------------Q-QQQETSP---PQQQ---QQQ
P15207/1-902 PPGACL----------------------Q-QRQETSP---RRRR---RQQ
G1TTV8/1-901 PPGARL--------QQQQQQQQQQQQQQQ-QQQETSP---RQQQ---QQQ
P19091/1-899 PPGACL----------------------Q-QRQETSP---RRRR---RQQ
G1PPI1/1-892 PPGAQL------------------------QQQQTSP---RQ-----QQQ
M3W7I8/1-903 PPGARL----------------QQQQQQQ-QQEQTSPQQQQQQQ---QQQ
Q9TT90/1-907 PPGAHL----------------QQQQQQQ-QQQETSP---RQQQ---QQQ
Q8MIK0/1-912 PPGARL--------------QQQHQHQQQ-HQHETSP---RRQ----QQQ
A0A3Q7SKY7/1-900 PPGAHL-------------QQQQQQQQQQ-QQQETSP---RQQQ---QQQ
F6R5G1/1-882 PPGAHL----------------------Q-QQQETSP-----RQ---QQQ
Q9GKL7/1-896 PPGARL-------------------QQQQLQQQETSP---RRQQQQ-QQQ
U4MHY9/1-894 PPGARL-------------------QQQQ-QQQETSP---RQQQQ--QQQ
S5U7M4/1-890 PPGARL----------------------Q-QQQETSP---RRQQQ--QQQ
F1N2B6/1-890 PSGARL----------------------Q-QQQETSP---RQQQQ--QQQ
A0A2Y9F3J7/1-893 PPGARL----------------------Q-QQQETSP---QQQQQQQQQQ
A0A2Y9M4V8/1-893 PPGARL----------------------Q-QQQETSP---QQQQQQRQQQ
A0A2U3V2H0/1-893 PPGARL----------------------Q-QQQETSP---QQQQQQRQQQ
A0A384ADZ0/1-895 PPGARL-------------------Q-QQ-QQQETSP---QQQQQQQQQQ
Q7T1K4/1-777 PISACV----------------------HPHPTWNEPSTWTEVR------
Conservation
*48*39----------------------1-654696*---0101---111
Quality
Consensus
PPGARLLLLQQQQQQQQQQQQQQQQQQQQ+QQQETSP+++RQQQQQ+QQQ
Occupancy

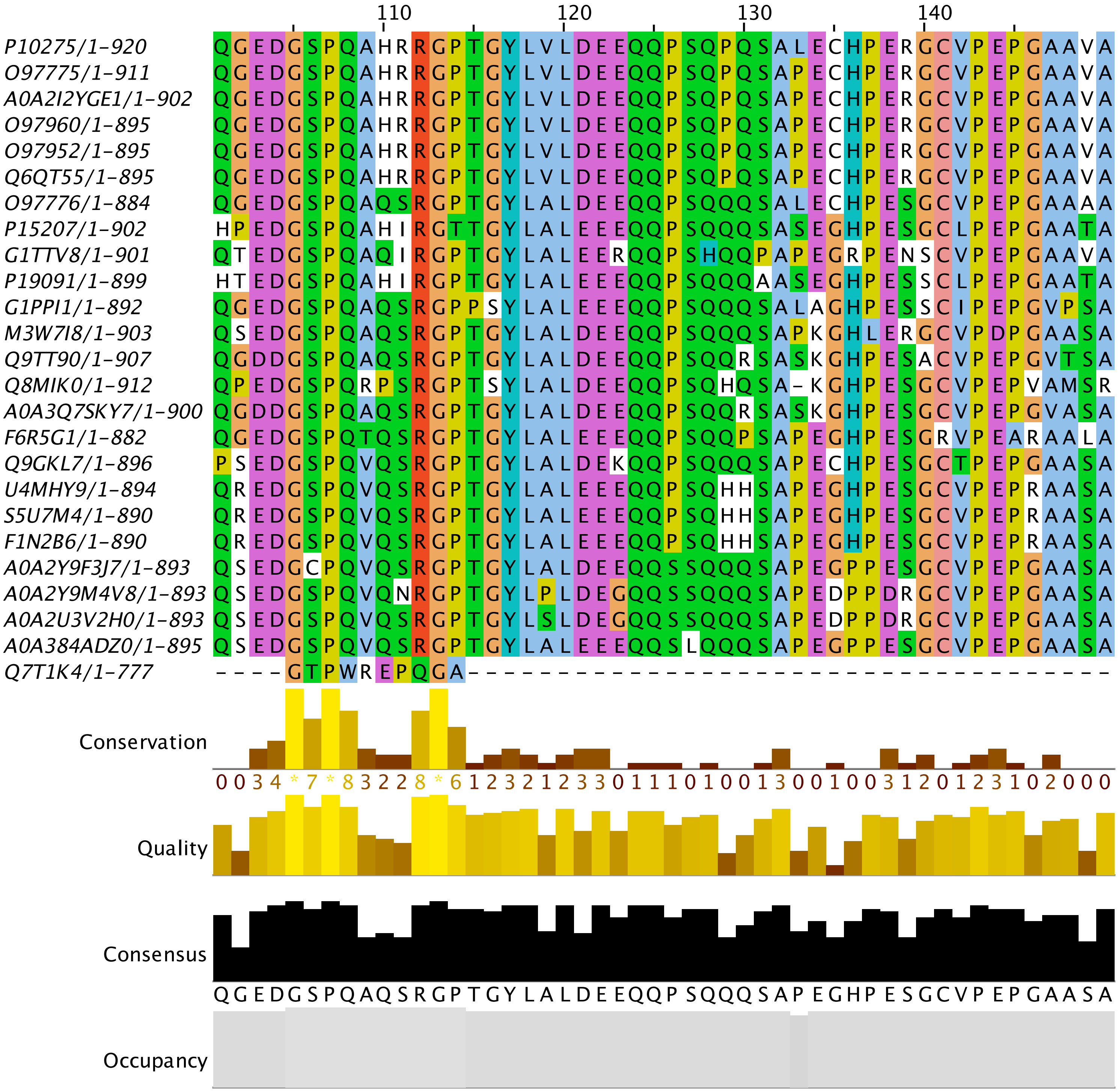

110 120 130 140
P10275/1-920 QGEDGSPQAHRRGPTGYLVLDEEQQPSQPQSALECHPERGCVPEPGAAVA
O97775/1-911 QGEDGSPQAHRRGPTGYLVLDEEQQPSQPQSAPECHPERGCVPEPGAAVA
A0A2I2YGE1/1-902 QGEDGSPQAHRRGPTGYLVLDEEQQPSQPQSAPECHPERGCVPEPGAAVA
O97960/1-895 QGEDGSPQAHRRGPTGYLVLDEEQQPSQPQSAPECHPERGCVPEPGAAVA
O97952/1-895 QGEDGSPQAHRRGPTGYLVLDEEQQPSQPQSAPECHPERGCVPEPGAAVA
Q6QT55/1-895 QGEDGSPQAHRRGPTGYLVLDEEQQPSQPQSAPECHPERGCVPEPGAAVA
O97776/1-884 QGEDGSPQAQSRGPTGYLALDEEQQPSQQQSALECHPESGCVPEPGAAAA
P15207/1-902 HPEDGSPQAHIRGTTGYLALEEEQQPSQQQSASEGHPESGCLPEPGAATA
G1TTV8/1-901 QTEDGSPQAQIRGPTGYLALEERQQPSHQQPAPEGRPENSCVPEPGAAVA
P19091/1-899 HTEDGSPQAHIRGPTGYLALEEEQQPSQQQAASEGHPESSCLPEPGAATA
G1PPI1/1-892 QGEDGSPQAQSRGPPSYLALEEEQQPSQQQSALAGHPESSCIPEPGVPSA
M3W7I8/1-903 QSEDGSPQAQSRGPTGYLALDEEQQPSQQQSAPKGHLERGCVPDPGAASA
Q9TT90/1-907 QGDDGSPQAQSRGPTGYLALDEEQQPSQQRSASKGHPESACVPEPGVTSA
Q8MIK0/1-912 QPEDGSPQRPSRGPTSYLALDEEQQPSQHQSA-KGHPESGCVPEPVAMSR
A0A3Q7SKY7/1-900 QGDDGSPQAQSRGPTGYLALDEEQQPSQQRSASKGHPESGCVPEPGVASA
F6R5G1/1-882 QGEDGSPQTQSRGPTGYLALEEEQQPSQQPSAPEGHPESGRVPEARAALA
Q9GKL7/1-896 PSEDGSPQVQSRGPTGYLALDEKQQPSQQQSAPECHPESGCTPEPGAASA
U4MHY9/1-894 QREDGSPQVQSRGPTGYLALEEEQQPSQHHSAPEGHPESGCVPEPRAASA
S5U7M4/1-890 QREDGSPQVQSRGPTGYLALEEEQQPSQHHSAPEGHPESGCVPEPRAASA
F1N2B6/1-890 QREDGSPQVQSRGPTGYLALEEEQQPSQHHSAPEGHPESGCVPEPRAASA
A0A2Y9F3J7/1-893 QSEDGCPQVQSRGPTGYLALDEEQQSSQQQSAPEGPPESGCVPEPGAASA
A0A2Y9M4V8/1-893 QSEDGSPQVQNRGPTGYLPLDEGQQSSQQQSAPEDPPDRGCVPEPGAASA
A0A2U3V2H0/1-893 QSEDGSPQVQSRGPTGYLSLDEGQQSSQQQSAPEDPPDRGCVPEPGAASA
A0A384ADZ0/1-895 QSEDGSPQVQSRGPTGYLALEEEQQSLQQQSAPEGPPESGCVPEPGAASA
Q7T1K4/1-777 ----GTPWREPQGA------------------------------------
Conservation
0 0 3 4 * 7 * 8 3 2 2 8 * 6 1 2 3 2 1 2 3 3 0 1 1 1 0 1 0 0 1 3 0 0 1 0 0 3 1 2 0 1 2 3 1 0 2 0 0 0
Quality
Consensus
QGEDGSPQAQSRGPTGYLALDEEQQPSQQQSAPEGHPESGCVPEPGAASA
Occupancy

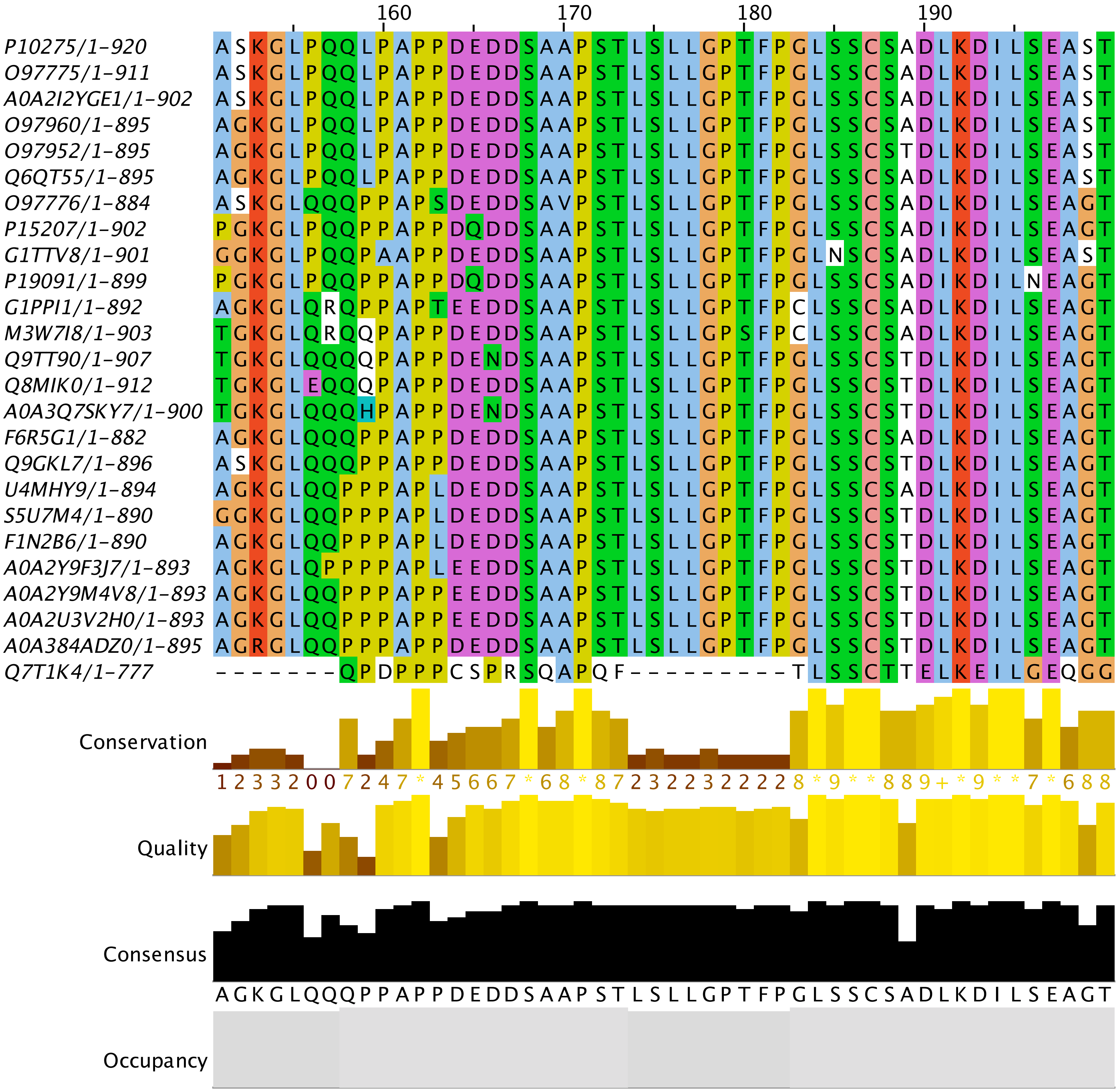
160
170
180
190
P10275/1-920
O97775/1-911
A0A2I2YGE1/1-902
O97960/1-895
O97952/1-895
Q6QT55/1-895
O97776/1-884
P15207/1-902
G1TTV8/1-901
P19091/1-899
G1PPI1/1-892
M3W7I8/1-903
Q9TT90/1-907
Q8MIK0/1-912
A0A3Q7SKY7/1-900
F6R5G1/1-882
Q9GKL7/1-896
U4MHY9/1-894
S5U7M4/1-890
F1N2B6/1-890
A0A2Y9F3J7/1-893
A0A2Y9M4V8/1-893
A0A2U3V2H0/1-893
A0A384ADZ0/1-895
Q7T1K4/1-777
Conservation
Quality
Consensus
Occupancy

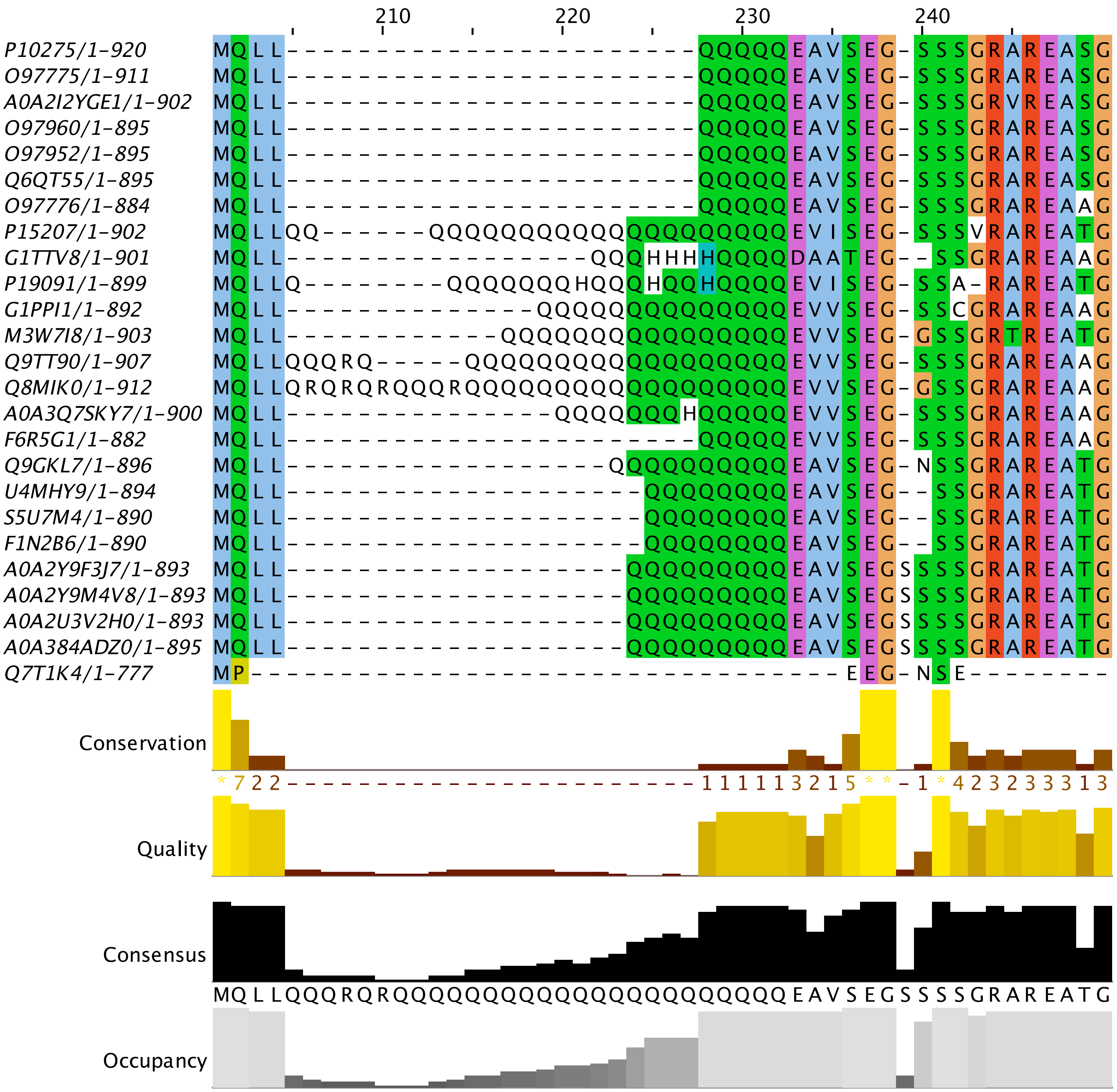

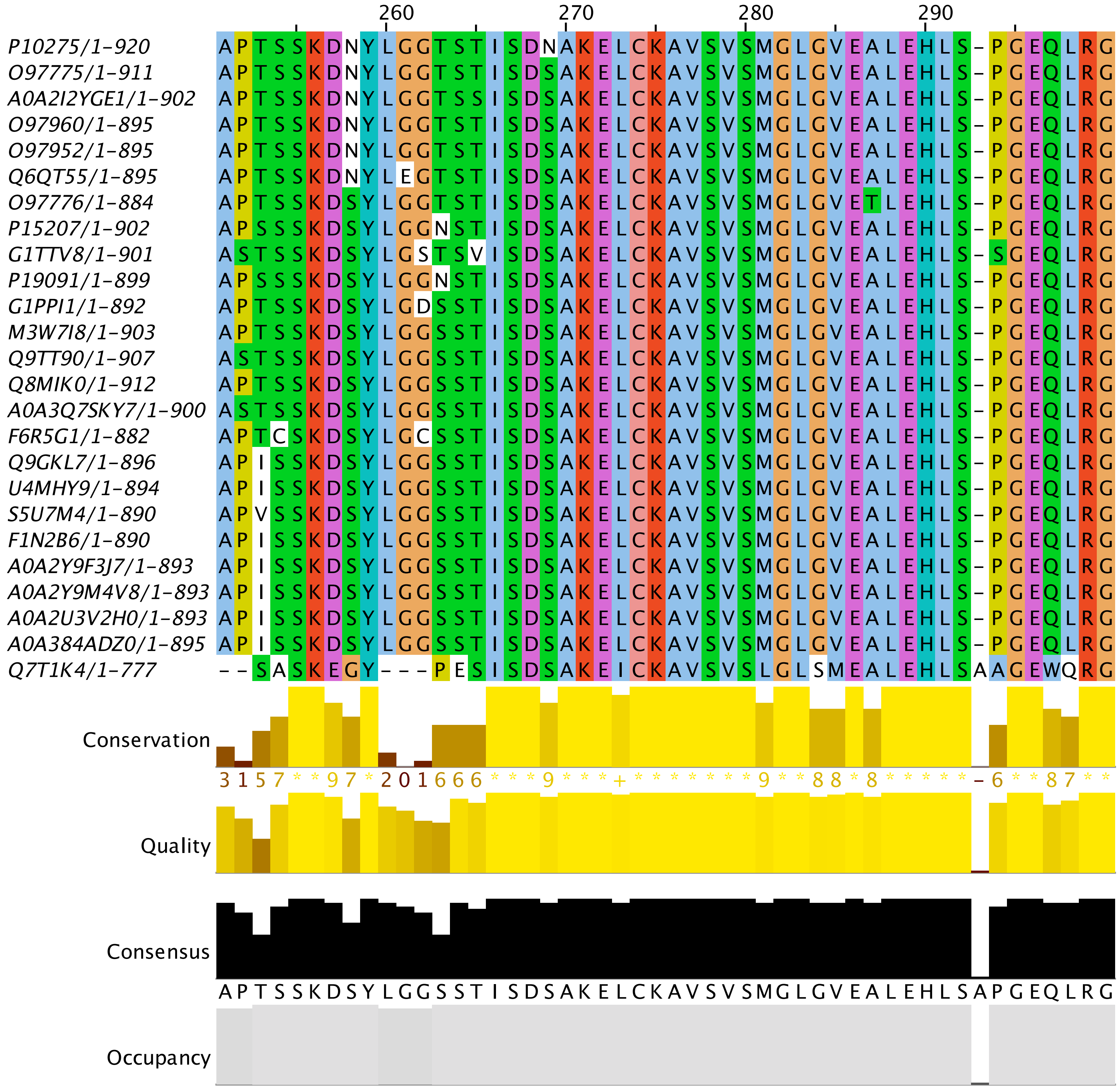
260 270 280 290
P10275/1-920 APTSSKDNYLGGTSTISDNAKELCKAVSVSMGLGVEALEHLS-PGEQLRG
O97775/1-911 APTSSKDNYLGGTSTISDSAKELCKAVSVSMGLGVEALEHLS-PGEQLRG
A0A2I2YGE1/1-902 APTSSKDNYLGGTSSISDSAKELCKAVSVSMGLGVEALEHLS-PGEQLRG
O97960/1-895 APTSSKDNYLGGTSTISDSAKELCKAVSVSMGLGVEALEHLS-PGEQLRG
O97952/1-895 APTSSKDNYLGGTSTISDSAKELCKAVSVSMGLGVEALEHLS-PGEQLRG
Q6QT55/1-895 APTSSKDNYLEGTSTISDSAKELCKAVSVSMGLGVEALEHLS-PGEQLRG
O97776/1-884 APTSSKDSYLGGTSTISDSAKELCKAVSVSMGLGVETLEHLS-PGEQLRG
P15207/1-902 APSSSKDSYLGGNSTISDSAKELCKAVSVSMGLGVEALEHLS-PGEQLRG
G1TTV8/1-901 ASTSSKDSYLGSTSVISDSAKELCKAVSVSMGLGVEALEHLS-SGEQLRG
P19091/1-899 APSSSKDSYLGGNSTISDSAKELCKAVSVSMGLGVEALEHLS-PGEQLRG
G1PPI1/1-892 APTSSKDSYLGDSSTISDSAKELCKAVSVSMGLGVEALEHLS-PGEQLRG
M3W7I8/1-903 APTSSKDSYLGGSSTISDSAKELCKAVSVSMGLGVEALEHLS-PGEQLRG
Q9TT90/1-907 ASTSSKDSYLGGSSTISDSAKELCKAVSVSMGLGVEALEHLS-PGEQLRG
Q8MIK0/1-912 APTSSKDSYLGGSSTISDSAKELCKAVSVSMGLGVEALEHLS-PGEQLRG
A0A3Q7SKY7/1-900 ASTSSKDSYLGGSSTISDSAKELCKAVSVSMGLGVEALEHLS-PGEQLRG
F6R5G1/1-882 APTCSKDSYLGCSSTISDSAKELCKAVSVSMGLGVEALEHLS-PGEQLRG
Q9GKL7/1-896 APISSKDSYLGGSSTISDSAKELCKAVSVSMGLGVEALEHLS-PGEQLRG
U4MHY9/1-894 APISSKDSYLGGSSTISDSAKELCKAVSVSMGLGVEALEHLS-PGEQLRG
S5U7M4/1-890 APVSSKDSYLGGSSTISDSAKELCKAVSVSMGLGVEALEHLS-PGEQLRG
F1N2B6/1-890 APISSKDSYLGGSSTISDSAKELCKAVSVSMGLGVEALEHLS-PGEQLRG
A0A2Y9F3J7/1-893 APISSKDSYLGGSSTISDSAKELCKAVSVSMGLGVEALEHLS-PGEQLRG
A0A2Y9M4V8/1-893 APISSKDSYLGGSSTISDSAKELCKAVSVSMGLGVEALEHLS-PGEQLRG
A0A2U3V2H0/1-893 APISSKDSYLGGSSTISDSAKELCKAVSVSMGLGVEALEHLS-PGEQLRG
A0A384ADZ0/1-895 APISSKDSYLGGSSTISDSAKELCKAVSVSMGLGVEALEHLS-PGEQLRG
Q7T1K4/1-777 --SASKEGY---PESISDSAKEICKAVSVSLGLSMEALEHLSAAGEWQRG
Conservation
3157**97*201666***9***+*******9**88*8*****-6**87**
Quality
Consensus
APTSSKDSYLGGSSTISDSAKELCKAVSVSMGLGVEALEHLSAPGEQLRG
Occupancy

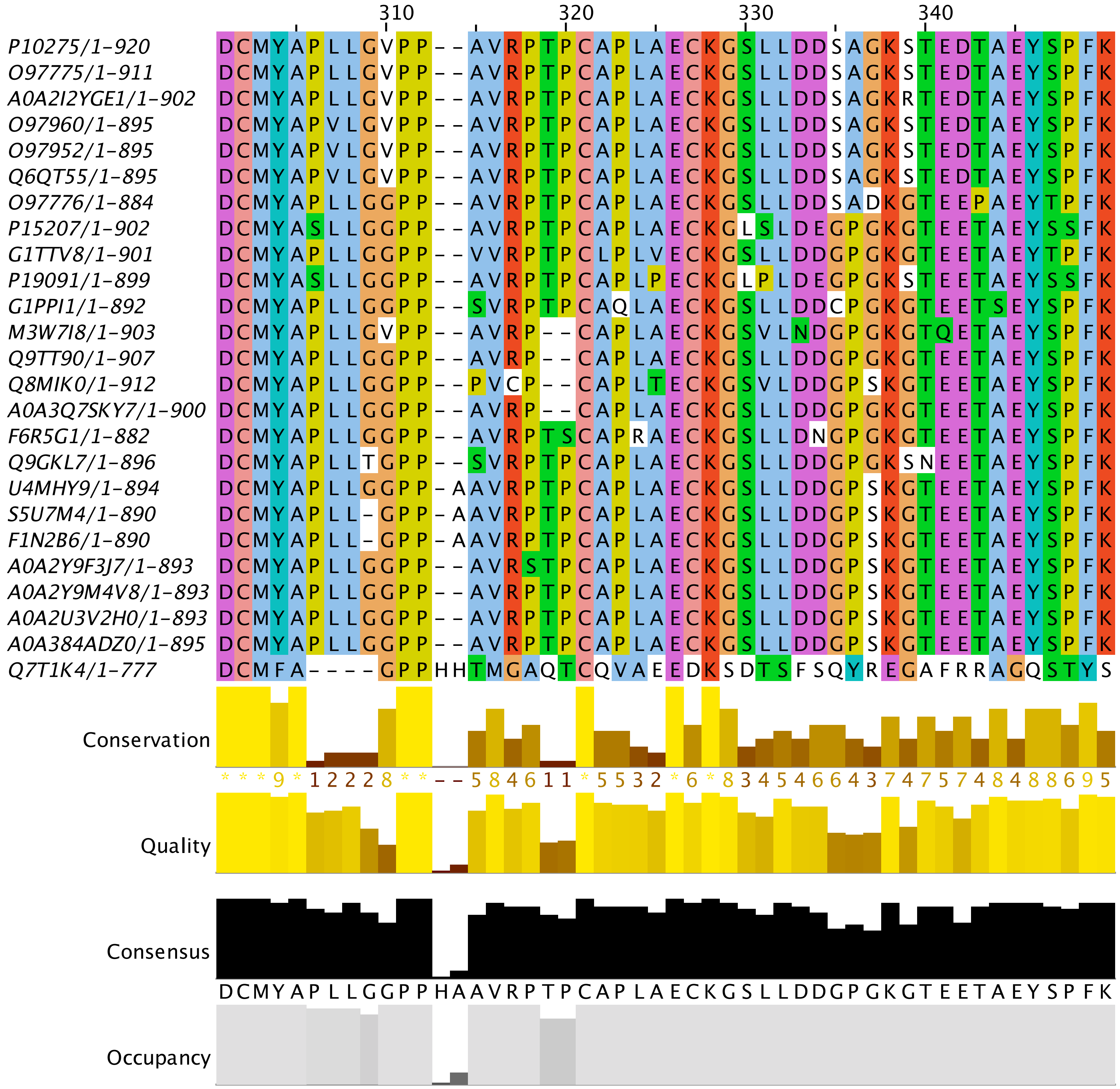
310
320
330
340
P10275/1-920 DCMYAPLLGVPP--AVRPTPCAPLAECKGSLLDDSAGKSTEDTAEYSPFK
O97775/1-911 DCMYAPLLGVPP--AVRPTPCAPLAECKGSLLDDSAGKSTEDTAEYSPFK
A0A2I2YGE1/1-902 DCMYAPLLGVPP--AVRPTPCAPLAECKGSLLDDSAGKRTEDTAEYSPFK
O97960/1-895 DCMYAPVLGVPP--AVRPTPCAPLAECKGSLLDDSAGKSTEDTAEYSPFK
O97952/1-895 DCMYAPVLGVPP--AVRPTPCAPLAECKGSLLDDSAGKSTEDTAEYSPFK
Q6QT55/1-895 DCMYAPVLGVPP--AVRPTPCAPLAECKGSLLDDSAGKSTEDTAEYSPFK
O97776/1-884 DCMYAPLLGGPP--AVRPTPCAPLAECKGSLLDDSADKGTEEPAEYTPFK
P15207/1-902 DCMYASLLGGPP--AVRPTPCAPLAECKGLSLDEGPGKGTEETAEYSSFK
G1TTV8/1-901 DCMYAPLLGGPP--VVRPTPCLPLVECKGSLLDDGPGKGTEETAEYTPFK
P19091/1-899 DCMYASLLGGPP--AVRPTPCAPLPECKGLPLDEGPGKSTEETAEYSSFK
G1PPI1/1-892 DCMYAPLLGGPP--SVRPTPCAQLAECKGSLLDDCPGKGTEETSEYSPFK
M3W7I8/1-903 DCMYAPLLGVPP--AVRP--CAPLAECKGSVLNDGPGKGTQETAEYSPFK
Q9TT90/1-907 DCMYAPLLGGPP--AVRP--CAPLAECKGSLLDDGPGKGTEETAEYSPFK
Q8MIK0/1-912 DCMYAPLLGGPP--PVCP--CAPLTECKGSVLDDGPSKGTEETAEYSPFK
A0A3Q7SKY7/1-900 DCMYAPLLGGPP--AVRP--CAPLAECKGSLLDDGPGKGTEETAEYSPFK
F6R5G1/1-882 DCMYAPLLGGPP--AVRPTSCAPRAECKGSLLDNGPGKGTEETAEYSPFK
Q9GKL7/1-896 DCMYAPLLTGPP--SVRPTPCAPLAECKGSLLDDGPGKSNEETAEYSPFK
U4MHY9/1-894 DCMYAPLLGGPP-AAVRPTPCAPLAECKGSLLDDGPSKGTEETAEYSPFK
S5U7M4/1-890 DCMYAPLL-GPP-AAVRPTPCAPLAECKGSLLDDGPSKGTEETAEYSPFK
F1N2B6/1-890 DCMYAPLL-GPP-AAVRPTPCAPLAECKGSLLDDGPSKGTEETAEYSPFK
A0A2Y9F3J7/1-893 DCMYAPLLGGPP--AVRSTPCAPLAECKGSLLDDGPSKGTEETAEYSPFK
A0A2Y9M4V8/1-893 DCMYAPLLGGPP--AVRPTPCAPLAECKGSLLDDGPSKGTEETAEYSPFK
A0A2U3V2H0/1-893 DCMYAPLLGGPP--AVRPTPCAPLAECKGSLLDDGPSKGTEETAEYSPFK
A0A384ADZ0/1-895 DCMYAPLLGGPP--AVRPTPCAPLAECKGSLLDDGPSKGTEETAEYSPFK
Q7T1K4/1-777 DCMFA----GPPHHTMGAQTCQVAEEDKSDTSFSQYREGAFRRAGQSTYS
Conservation
***9*12228**--584611*5532*6*8345466437475748488695
Quality
Consensus
DCMYAPLLGGPPHAAVRPTPCAPLAECKGSLLDDGPGKGTEETAEYSPFK
Occupancy

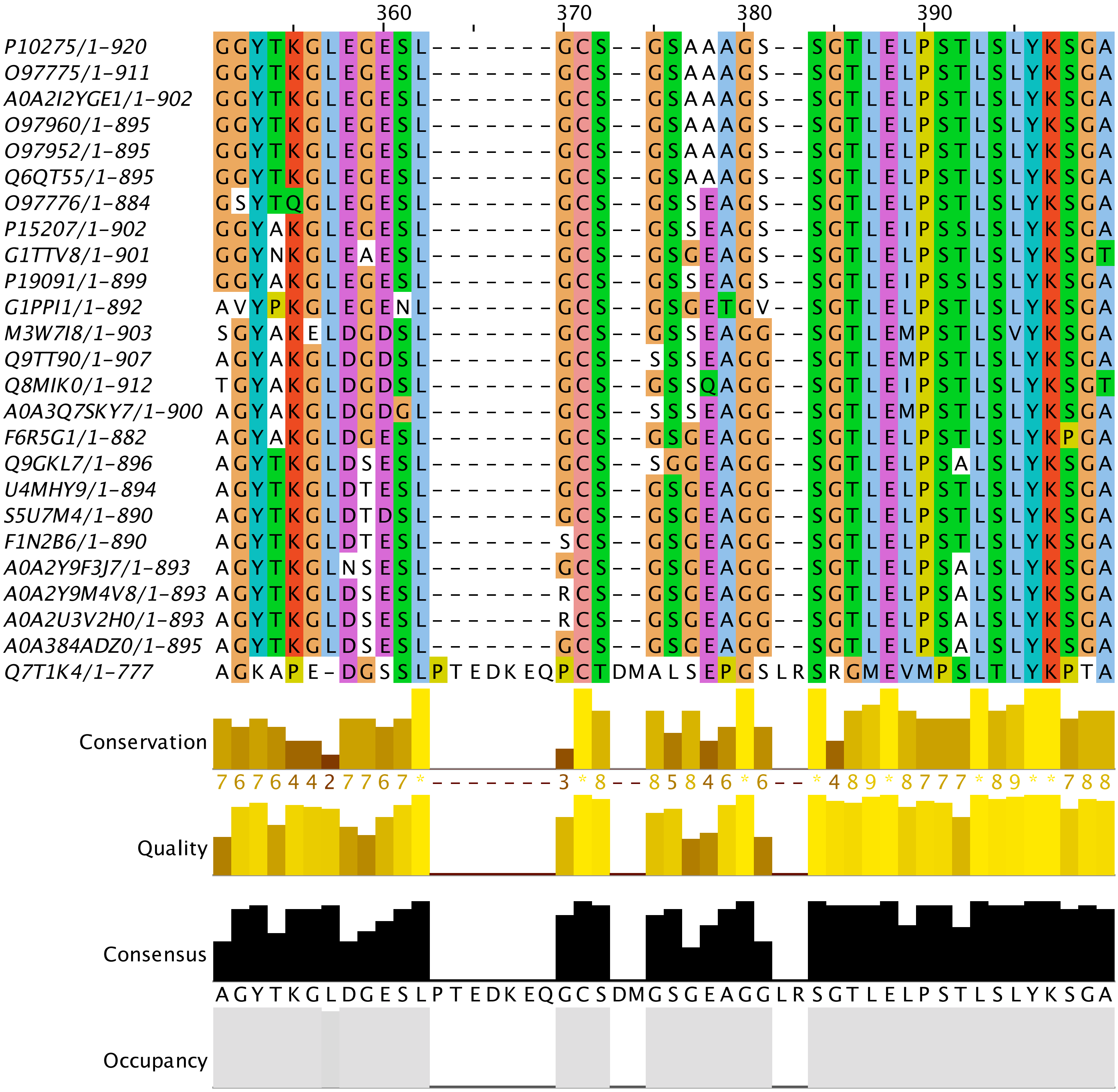
360
370
380
390
P10275/1-920
O97775/1-911
A0A2I2YGE1/1-902
O97960/1-895
O97952/1-895
Q6QT55/1-895
O97776/1-884
P15207/1-902
G1TTV8/1-901
P19091/1-899
G1PPI1/1-892
M3W7I8/1-903
Q9TT90/1-907
Q8MIK0/1-912
A0A3Q7SKY7/1-900
F6R5G1/1-882
Q9GKL7/1-896
U4MHY9/1-894
S5U7M4/1-890
F1N2B6/1-890
A0A2Y9F3J7/1-893
A0A2Y9M4V8/1-893
A0A2U3V2H0/1-893
A0A384ADZ0/1-895
Q7T1K4/1-777
Conservation
Quality
Consensus
AGYTKGLDGESLPTEDKEQGCSDMGSGEAGGLRSGTLELPSTLSLYKSGA
Occupancy

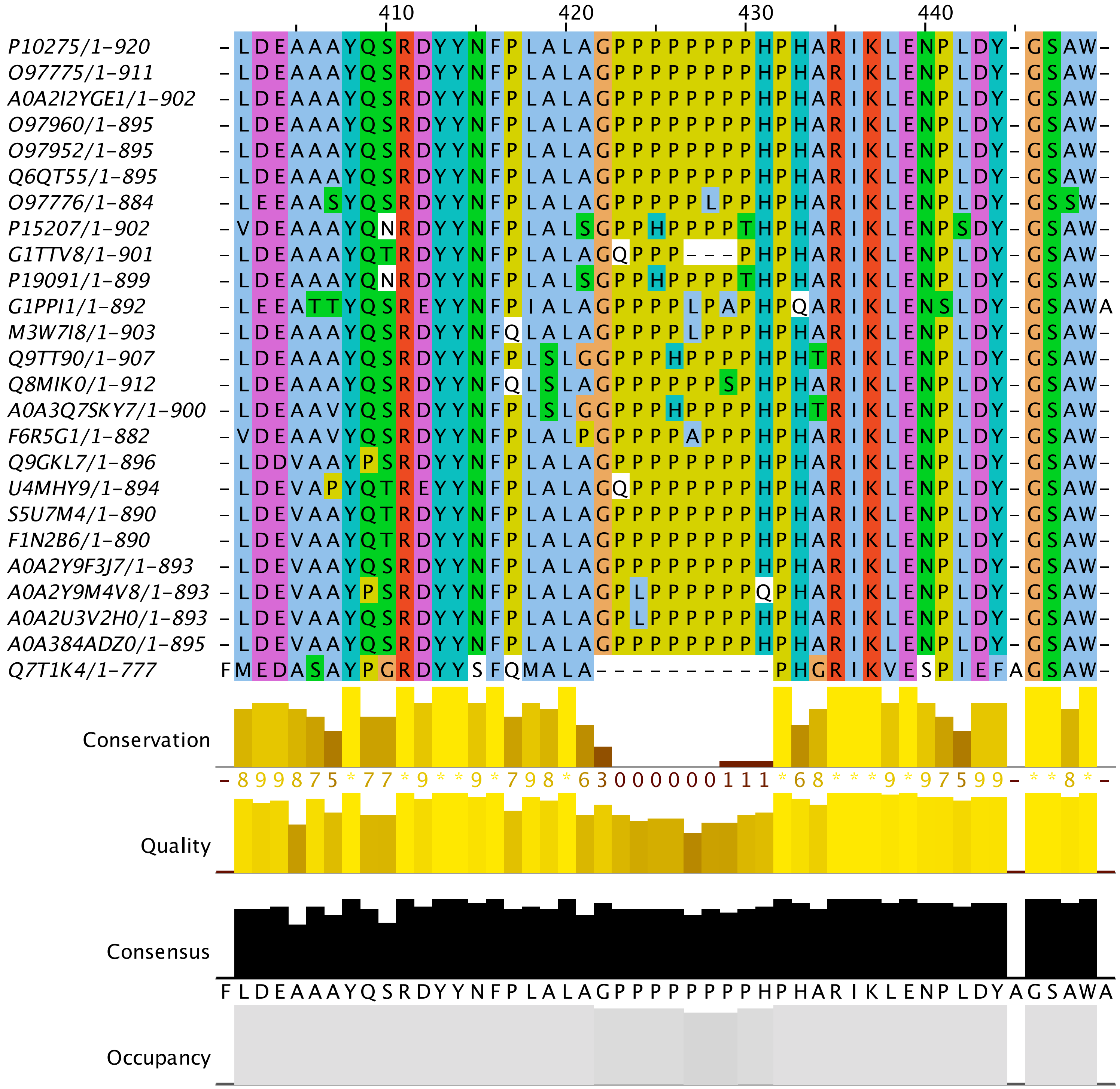

410
420
430
440
P10275/1-920 -LDEAAAYQSRDYYNFPLALAGPPPPPPPPHPHARIKLENPLDY-GSAW-
O97775/1-911 -LDEAAAYQSRDYYNFPLALAGPPPPPPPPHPHARIKLENPLDY-GSAW-
A0A2I2YGE1/1-902 -LDEAAAYQSRDYYNFPLALAGPPPPPPPPHPHARIKLENPLDY-GSAW-
O97960/1-895 -LDEAAAYQSRDYYNFPLALAGPPPPPPPPHPHARIKLENPLDY-GSAW-
O97952/1-895 -LDEAAAYQSRDYYNFPLALAGPPPPPPPPHPHARIKLENPLDY-GSAW-
Q6QT55/1-895 -LDEAAAYQSRDYYNFPLALAGPPPPPPPPHPHARIKLENPLDY-GSAW-
O97776/1-884 -LEEAASYQSRDYYNFPLALAGPPPPPLPPHPHARIKLENPLDY-GSSW-
P15207/1-902 -VDEAAAYQNRDYYNFPLALSGPPHPPPPTHPHARIKLENPSDY-GSAW-
G1TTV8/1-901 -LDEAAAYQTRDYYNFPLALAGQPPP---PHPHARIKLENPLDY-GSAW-
P19091/1-899 -LDEAAAYQNRDYYNFPLALSGPPHPPPPTHPHARIKLENPLDY-GSAW-
G1PPI1/1-892 -LEEATTYQSREYYNFPIALAGPPPPLPAPHPQARIKLENSLDY-GSAWA
M3W7I8/1-903 -LDEAAAYQSRDYYNFQLALAGPPPPLPPPHPHARIKLENPLDY-GSAW-
Q9TT90/1-907 -LDEAAAYQSRDYYNFPLSLGGPPPHPPPPHPHTRIKLENPLDY-GSAW-
Q8MIK0/1-912 -LDEAAAYQSRDYYNFQLSLAGPPPPPPSPHPHARIKLENPLDY-GSAW-
A0A3Q7SKY7/1-900 -LDEAAVYQSRDYYNFPLSLGGPPPHPPPPHPHTRIKLENPLDY-GSAW-
F6R5G1/1-882 -VDEAAVYQSRDYYNFPLALPGPPPPAPPPHPHARIKLENPLDY-GSAW-
Q9GKL7/1-896 -LDDVAAYPSRDYYNFPLALAGPPPPPPPPHPHARIKLENPLDY-GSAW-
U4MHY9/1-894 -LDEVAPYQTREYYNFPLALAGQPPPPPPPHPHARIKLENPLDY-GSAW-
S5U7M4/1-890 -LDEVAAYQTRDYYNFPLALAGPPPPPPPPHPHARIKLENPLDY-GSAW-
F1N2B6/1-890 -LDEVAAYQTRDYYNFPLALAGPPPPPPPPHPHARIKLENPLDY-GSAW-
A0A2Y9F3J7/1-893 -LDEVAAYQSRDYYNFPLALAGPPPPPPPPHPHARIKLENPLDY-GSAW-
A0A2Y9M4V8/1-893 -LDEVAAYPSRDYYNFPLALAGPLPPPPPPQPHARIKLENPLDY-GSAW-
A0A2U3V2H0/1-893 -LDEVAAYQSRDYYNFPLALAGPLPPPPPPHPHARIKLENPLDY-GSAW-
A0A384ADZ0/1-895 -LDEVAAYQSRDYYNFPLALAGPPPPPPPPHPHARIKLENPLDY-GSAW-
Q7T1K4/1-777 FMEDASAYPGRDYYSFQMALA----------PHGRIKVESPIEFAGSAW-
Conservation
-899875*77*9**9*798*63000000111*68***9*97599-**8*-
Quality
Consensus
FLDEAAAYQSRDYYNFPLALAGPPPPPPPPHPHARIKLENPLDYAGSAWA
Occupancy

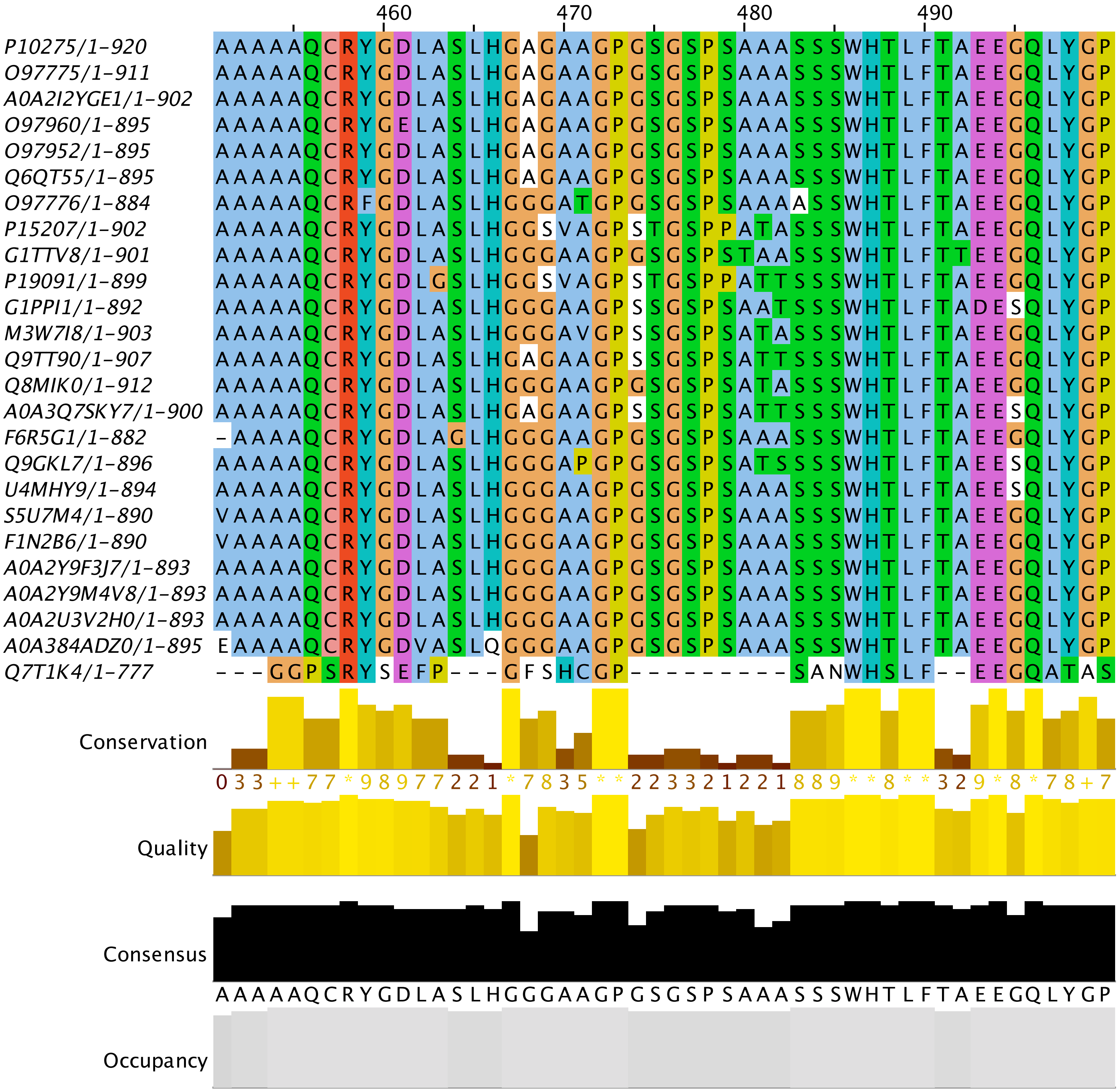
460
470
480
490
P10275/1-920 AAAAAQCRYGDLASLHGAGAAGPGSGSPSAAASSSWHTLFTAEEGQLYGP
O97775/1-911 AAAAAQCRYGDLASLHGAGAAGPGSGSPSAAASSSWHTLFTAEEGQLYGP
A0A2I2YGE1/1-902 AAAAAQCRYGDLASLHGAGAAGPGSGSPSAAASSSWHTLFTAEEGQLYGP
O97960/1-895 AAAAAQCRYGELASLHGAGAAGPGSGSPSAAASSSWHTLFTAEEGQLYGP
O97952/1-895 AAAAAQCRYGDLASLHGAGAAGPGSGSPSAAASSSWHTLFTAEEGQLYGP
Q6QT55/1-895 AAAAAQCRYGDLASLHGAGAAGPGSGSPSAAASSSWHTLFTAEEGQLYGP
O97776/1-884 AAAAAQCRFGDLASLHGGGATGPGSGSPSAAAASSWHTLFTAEEGQLYGP
P15207/1-902 AAAAAQCRYGDLASLHGGSVAGPSTGSPPATASSSWHTLFTAEEGQLYGP
G1TTV8/1-901 AAAAAQCRYGDLASLHGGGAAGPGSGSPSTAASSSWHTLFTTEEGQLYGP
P19091/1-899 AAAAAQCRYGDLGSLHGGSVAGPSTGSPPATTSSSWHTLFTAEEGQLYGP
G1PPI1/1-892 AAAAAQCRYGDLASLHGGGAAGPSSGSPSAATSSSWHTLFTADESQLYGP
M3W7I8/1-903 AAAAAQCRYGDLASLHGGGAVGPSSGSPSATASSSWHTLFTAEEGQLYGP
Q9TT90/1-907 AAAAAQCRYGDLASLHGAGAAGPSSGSPSATTSSSWHTLFTAEEGQLYGP
Q8MIK0/1-912 AAAAAQCRYGDLASLHGGGAAGPGSGSPSATASSSWHTLFTAEEGQLYGP
A0A3Q7SKY7/1-900 AAAAAQCRYGDLASLHGAGAAGPSSGSPSATTSSSWHTLFTAEESQLYGP
F6R5G1/1-882 -AAAAQCRYGDLAGLHGGGAAGPGSGSPSAAASSSWHTLFTAEEGQLYGP
Q9GKL7/1-896 AAAAAQCRYGDLASLHGGGAPGPGSGSPSATSSSSWHTLFTAEESQLYGP
U4MHY9/1-894 AAAAAQCRYGDLASLHGGGAAGPGSGSPSAAASSSWHTLFTAEESQLYGP
S5U7M4/1-890 VAAAAQCRYGDLASLHGGGAAGPGSGSPSAAASSSWHTLFTAEEGQLYGP
F1N2B6/1-890 VAAAAQCRYGDLASLHGGGAAGPGSGSPSAAASSSWHTLFTAEEGQLYGP
A0A2Y9F3J7/1-893 AAAAAQCRYGDLASLHGGGAAGPGSGSPSAAASSSWHTLFTAEEGQLYGP
A0A2Y9M4V8/1-893 AAAAAQCRYGDLASLHGGGAAGPGSGSPSAAASSSWHTLFTAEEGQLYGP
A0A2U3V2H0/1-893 AAAAAQCRYGDLASLHGGGAAGPGSGSPSAAASSSWHTLFTAEEGQLYGP
A0A384ADZ0/1-895 EAAAAQCRYGDVASLQGGGAAGPGSGSPSAAASSSWHTLFTAEEGQLYGP
Q7T1K4/1-777 ---GGPSRYSEFP---GFSHCGP---------SANWHSLF--EEGQATAS
Conservation
0 3 3 + + 7 7 * 9 8 9 7 7 2 2 1 * 7 8 3 5 * * 2 2 3 3 2 1 2 2 1 8 8 9 * * 8 * * 3 2 9 * 8 * 7 8 + 7
Quality
Consensus
AAAAAQCRYGDLASLHGGGAAGPGSGSPSAAASSSWHTLFTAEEGQLYGP
Occupancy

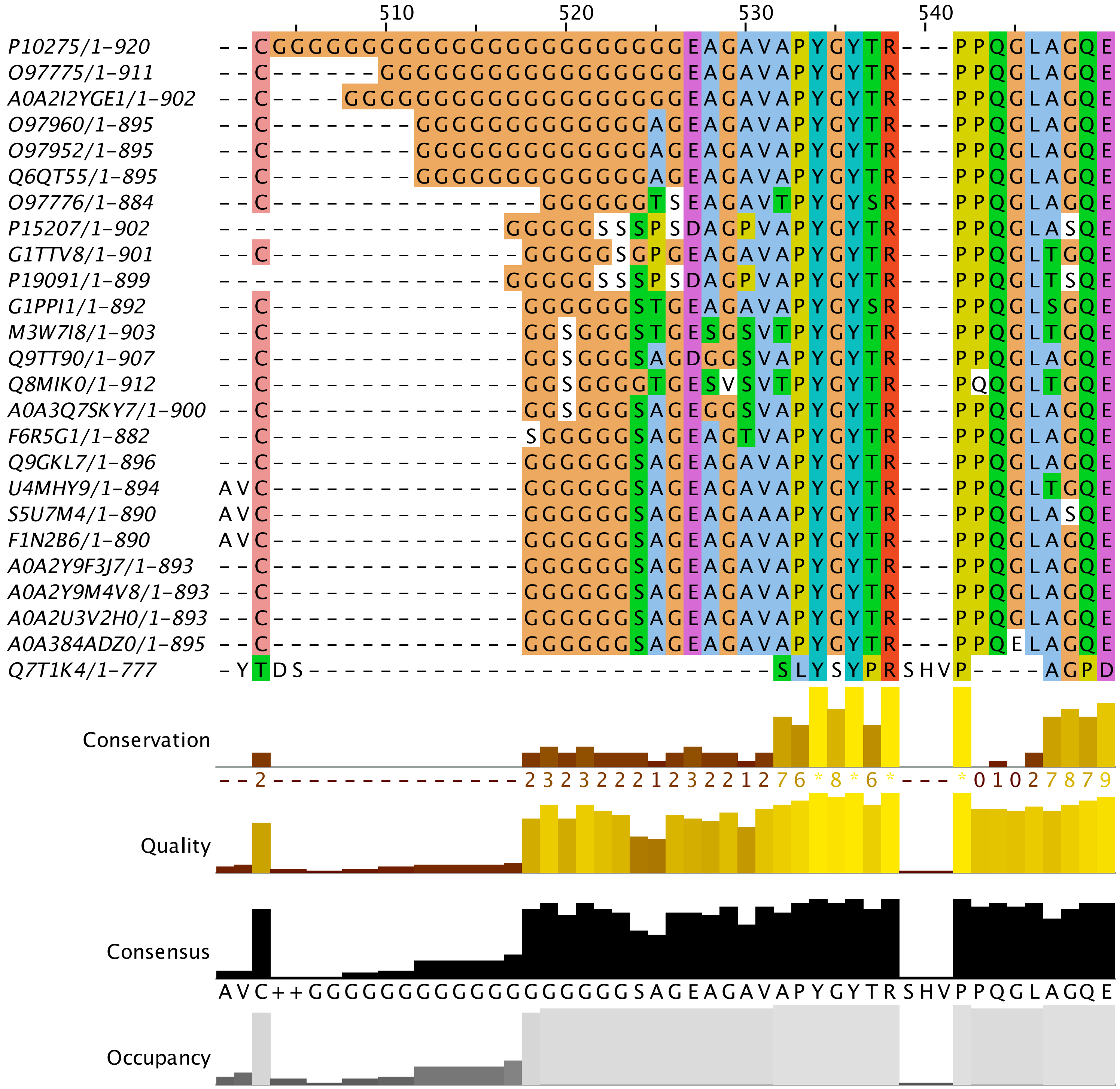

510
520
530
540
P10275/1-920
O97775/1-911
A0A2I2YGE1/1-902
O97960/1-895
O97952/1-895
Q6QT55/1-895
O97776/1-884
P15207/1-902
G1TTV8/1-901
P19091/1-899
G1PPI1/1-892
M3W7I8/1-903
Q9TT90/1-907
Q8MIK0/1-912
A0A3Q7SKY7/1-900
F6R5G1/1-882
Q9GKL7/1-896
U4MHY9/1-894
S5U7M4/1-890
F1N2B6/1-890
A0A2Y9F3J7/1-893
A0A2Y9M4V8/1-893
A0A2U3V2H0/1-893
A0A384ADZ0/1-895
Q7T1K4/1-777
Conservation
Quality
Consensus
AVC++GGGGGGGGGGGGGGGGGSAGEAGAVAPYGYTRSHVPPQGLAGQE
Occupancy

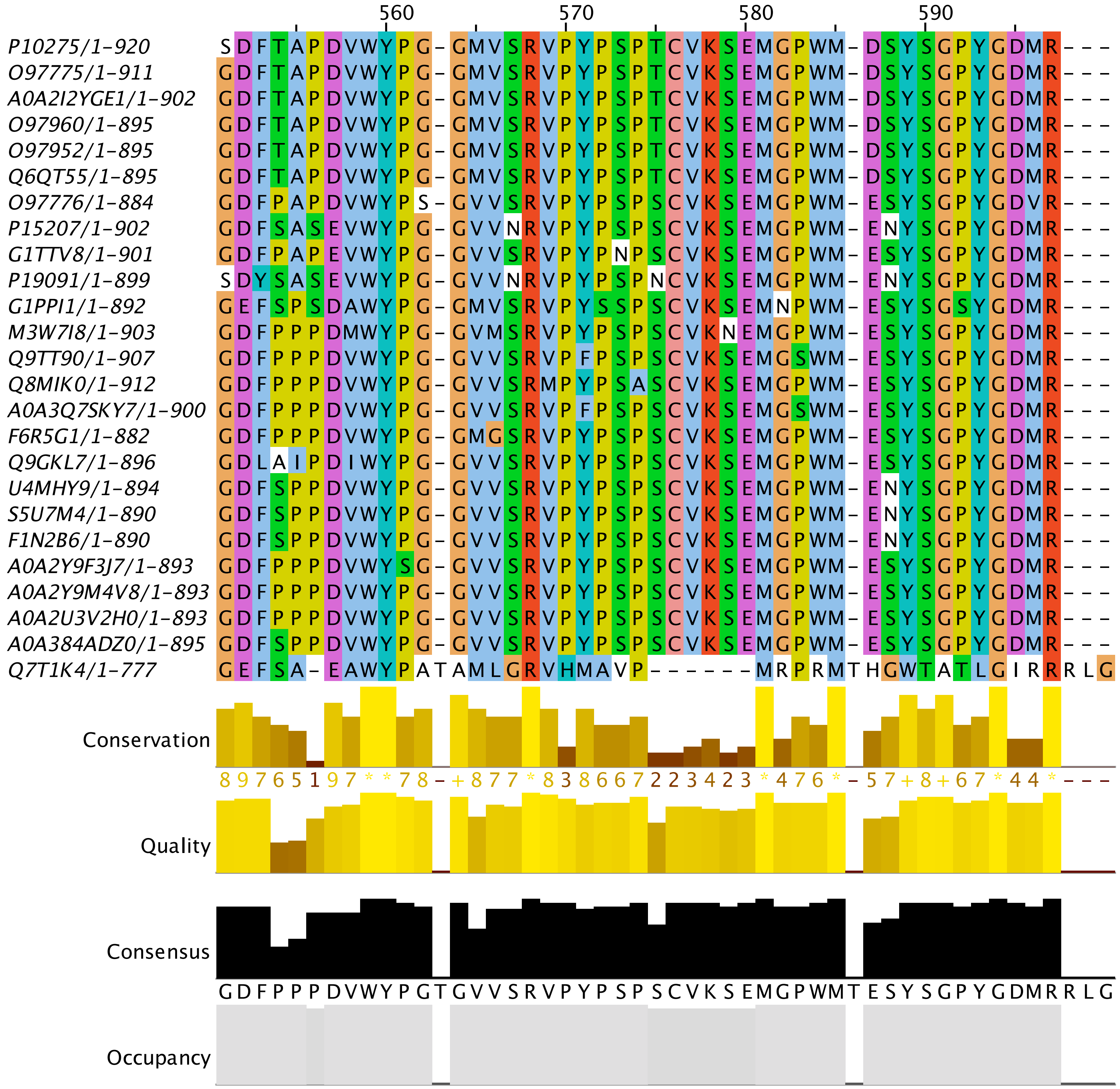
560
570
580
590
P10275/1-920 SDFTAPDVWYPG-GMVSRVPYPSPTCVKSEMGPWM-DSYSGPYGDMR---
O97775/1-911 GDFTAPDVWYPG-GMVSRVPYPSPTCVKSEMGPWM-DSYSGPYGDMR---
A0A2I2YGE1/1-902 GDFTAPDVWYPG-GMVSRVPYPSPTCVKSEMGPWM-DSYSGPYGDMR---
O97960/1-895 GDFTAPDVWYPG-GMVSRVPYPSPTCVKSEMGPWM-DSYSGPYGDMR---
O97952/1-895 GDFTAPDVWYPG-GMVSRVPYPSPTCVKSEMGPWM-DSYSGPYGDMR---
Q6QT55/1-895 GDFTAPDVWYPG-GMVSRVPYPSPTCVKSEMGPWM-DSYSGPYGDMR---
O97776/1-884 GDFPAPDVWYPS-GVVSRVPYPSPSCVKSEMGPWM-ESYSGPYGDVR---
P15207/1-902 GDFSASEVWYPG-GVVNRVPYPSPSCVKSEMGPWM-ENYSGPYGDMR---
G1TTV8/1-901 GDFPAPEVWYPG-GVVSRVPYPNPSCVKSEMGPWM-ESYSGPYGDMR---
P19091/1-899 SDYSASEVWYPG-GVVNRVPYPSPNCVKSEMGPWM-ENYSGPYGDMR---
G1PPI1/1-892 GEFSPSDAWYPG-GMVSRVPYSSPSCVKSEMNPWM-ESYSGSYGDMR---
M3W7I8/1-903 GDFPPPDMWYPG-GVMSRVPYPSPSCVKNEMGPWM-ESYSGPYGDMR---
Q9TT90/1-907 GDFPPPDVWYPG-GVVSRVPFPSPSCVKSEMGSWM-ESYSGPYGDMR---
Q8MIK0/1-912 GDFPPPDVWYPG-GVVSRMPYPSASCVKSEMGPWM-ESYSGPYGDMR---
A0A3Q7SKY7/1-900 GDFPPPDVWYPG-GVVSRVPFPSPSCVKSEMGSWM-ESYSGPYGDMR---
F6R5G1/1-882 GDFPPPDVWYPG-GMGSRVPYPSPSCVKSEMGPWM-ESYSGPYGDMR---
Q9GKL7/1-896 GDLAIPDIWYPG-GVVSRVPYPSPSCVKSEMGPWM-ESYSGPYGDMR---
U4MHY9/1-894 GDFSPPDVWYPG-GVVSRVPYPSPSCVKSEMGPWM-ENYSGPYGDMR---
S5U7M4/1-890 GDFSPPDVWYPG-GVVSRVPYPSPSCVKSEMGPWM-ENYSGPYGDMR---
F1N2B6/1-890 GDFSPPDVWYPG-GVVSRVPYPSPSCVKSEMGPWM-ENYSGPYGDMR---
A0A2Y9F3J7/1-893 GDFPPPDVWYSG-GVVSRVPYPSPSCVKSEMGPWM-ESYSGPYGDMR---
A0A2Y9M4V8/1-893 GDFPPPDVWYPG-GVVSRVPYPSPSCVKSEMGPWM-ESYSGPYGDMR---
A0A2U3V2H0/1-893 GDFPPPDVWYPG-GVVSRVPYPSPSCVKSEMGPWM-ESYSGPYGDMR---
A0A384ADZ0/1-895 GDFSPPDVWYPG-GVVSRVPYPSPSCVKSEMGPWM-ESYSGPYGDMR---
Q7T1K4/1-777 GEFSA-EAWYPATAMLGRVHMAVP------MRPRMTHGWTATLGIRRRLG
Conservation
8 9 7 6 5 1 9 7 * * 7 8 - + 8 7 7 * 8 3 8 6 6 7 2 2 3 4 2 3 * 4 7 6 * - 5 7 + 8 + 6 7 * 4 4 * - - -
Quality
Consensus
GDFPPPDVWYPGTGVVSRVPYPSPSCVKSEMGPWMTESYSGPYGDMRRLG
Occupancy

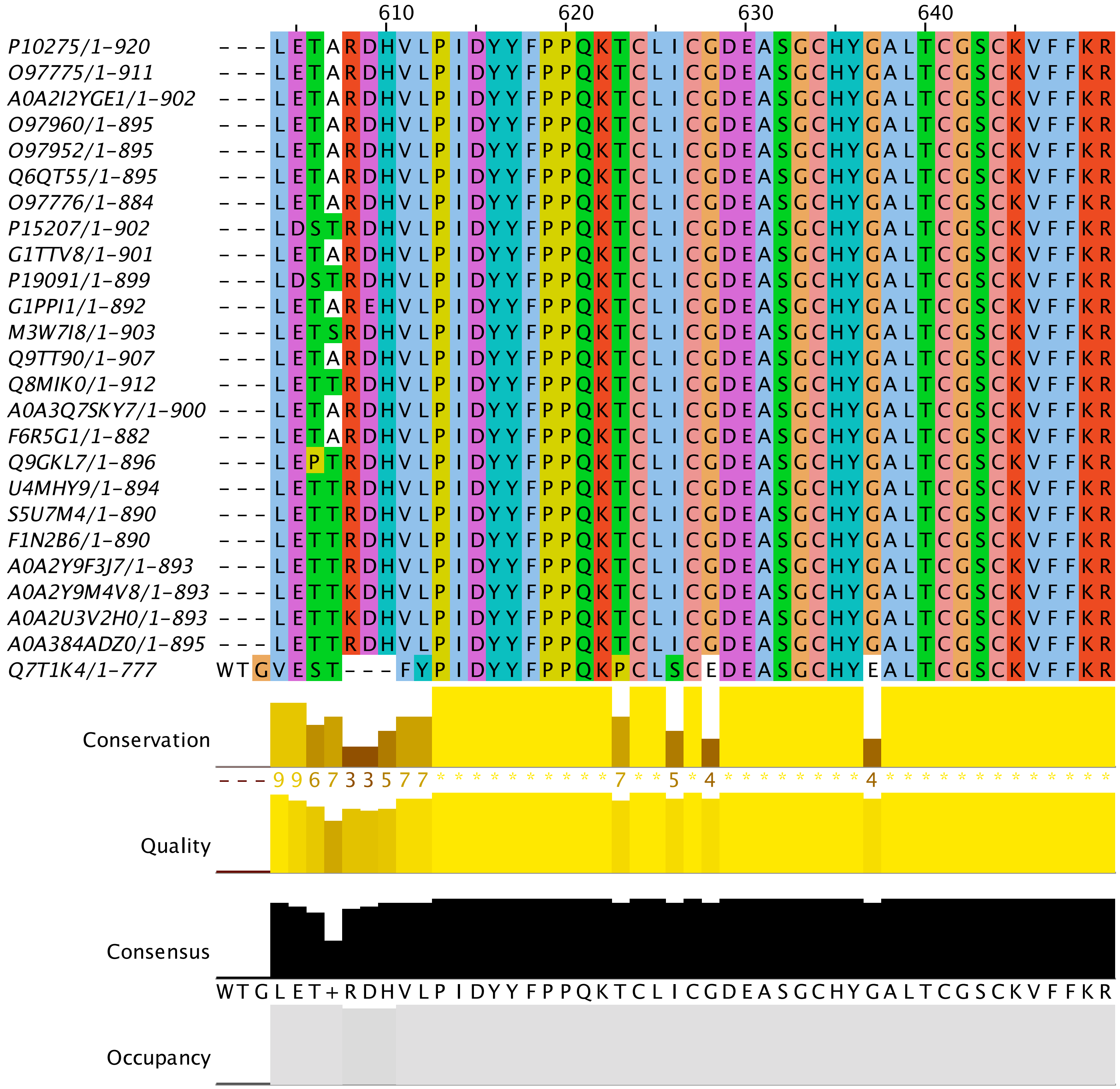

610 620 630 640
P10275/1-920 ---LETARDHVLPIDYYFPPQKTCLICGDEASGCHYGALTCGSCKVFFKR
O97775/1-911 ---LETARDHVLPIDYYFPPQKTCLICGDEASGCHYGALTCGSCKVFFKR
A0A2I2YGE1/1-902 ---LETARDHVLPIDYYFPPQKTCLICGDEASGCHYGALTCGSCKVFFKR
O97960/1-895 ---LETARDHVLPIDYYFPPQKTCLICGDEASGCHYGALTCGSCKVFFKR
O97952/1-895 ---LETARDHVLPIDYYFPPQKTCLICGDEASGCHYGALTCGSCKVFFKR
Q6QT55/1-895 ---LETARDHVLPIDYYFPPQKTCLICGDEASGCHYGALTCGSCKVFFKR
O97776/1-884 ---LETARDHVLPIDYYFPPQKTCLICGDEASGCHYGALTCGSCKVFFKR
P15207/1-902 ---LDSTRDHVLPIDYYFPPQKTCLICGDEASGCHYGALTCGSCKVFFKR
G1TTV8/1-901 ---LETARDHVLPIDYYFPPQKTCLICGDEASGCHYGALTCGSCKVFFKR
P19091/1-899 ---LDSTRDHVLPIDYYFPPQKTCLICGDEASGCHYGALTCGSCKVFFKR
G1PPI1/1-892 ---LETAREHVLPIDYYFPPQKTCLICGDEASGCHYGALTCGSCKVFFKR
M3W7I8/1-903 ---LETSRDHVLPIDYYFPPQKTCLICGDEASGCHYGALTCGSCKVFFKR
Q9TT90/1-907 ---LETARDHVLPIDYYFPPQKTCLICGDEASGCHYGALTCGSCKVFFKR
Q8MIK0/1-912 ---LETTRDHVLPIDYYFPPQKTCLICGDEASGCHYGALTCGSCKVFFKR
A0A3Q7SKY7/1-900 ---LETARDHVLPIDYYFPPQKTCLICGDEASGCHYGALTCGSCKVFFKR
F6R5G1/1-882 ---LETARDHVLPIDYYFPPQKTCLICGDEASGCHYGALTCGSCKVFFKR
Q9GKL7/1-896 ---LEPTRDHVLPIDYYFPPQKTCLICGDEASGCHYGALTCGSCKVFFKR
U4MHY9/1-894 ---LETTRDHVLPIDYYFPPQKTCLICGDEASGCHYGALTCGSCKVFFKR
S5U7M4/1-890 ---LETTRDHVLPIDYYFPPQKTCLICGDEASGCHYGALTCGSCKVFFKR
F1N2B6/1-890 ---LETTRDHVLPIDYYFPPQKTCLICGDEASGCHYGALTCGSCKVFFKR
A0A2Y9F3J7/1-893 ---LETTRDHVLPIDYYFPPQKTCLICGDEASGCHYGALTCGSCKVFFKR
A0A2Y9M4V8/1-893 ---LETTKDHVLPIDYYFPPQKTCLICGDEASGCHYGALTCGSCKVFFKR
A0A2U3V2H0/1-893 ---LETTKDHVLPIDYYFPPQKTCLICGDEASGCHYGALTCGSCKVFFKR
A0A384ADZ0/1-895 ---LETTRDHVLPIDYYFPPQKTCLICGDEASGCHYGALTCGSCKVFFKR
Q7T1K4/1-777 WTGVEST---FYPIDYYFPPQKPCLSCEDEASGCHYEALTCGSCKVFFKR
Conservation
---996733577**********7**5*4********4*************
Quality
Consensus
WTGLET+RDHVLPIDYYFPPQKTCLICGDEASGCHYGALTCGSCKVFFKR
Occupancy

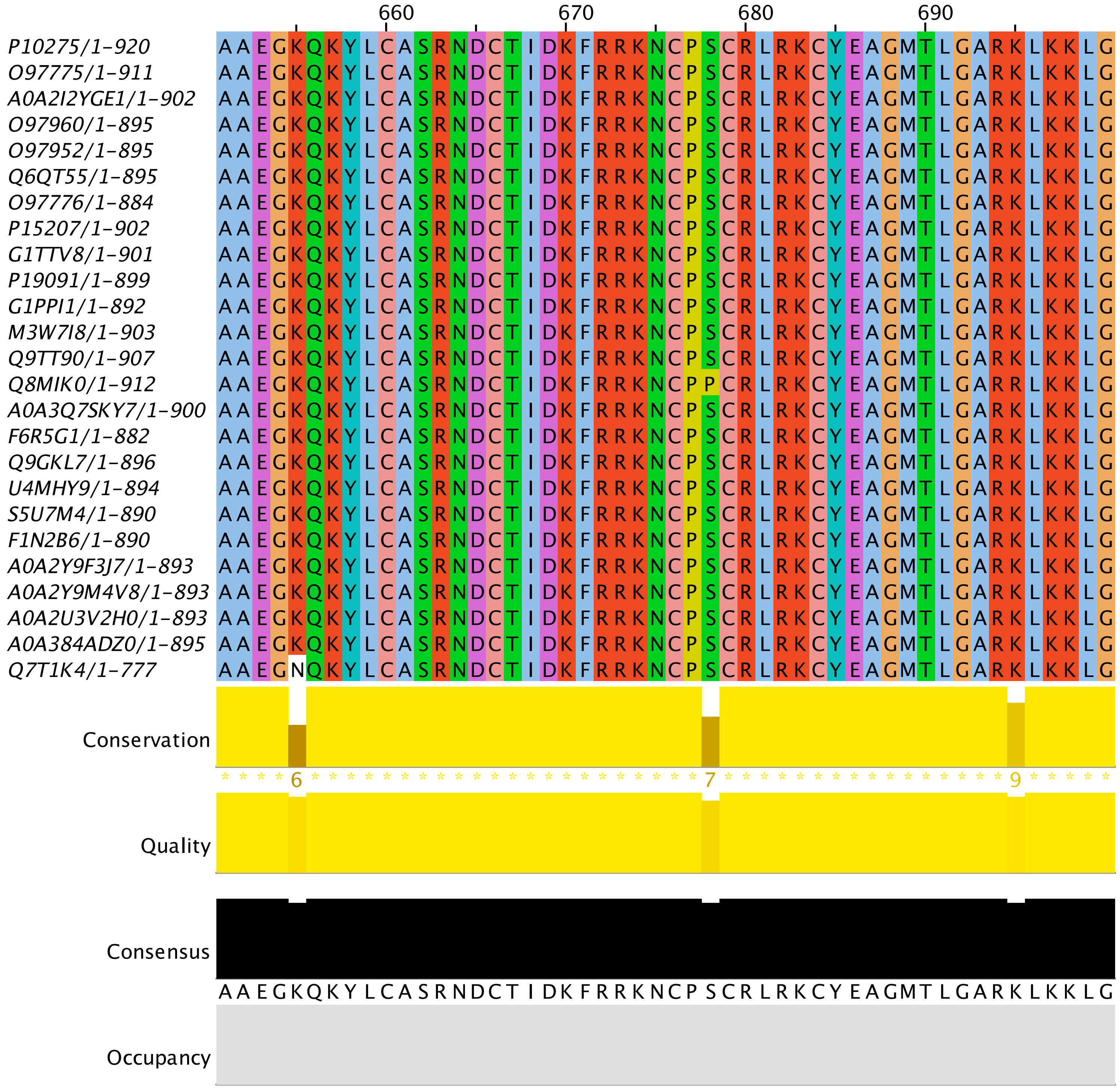
660
670
680
690
P10275/1-920 AAEGKQKYLCASRNDCTIDKFRRKNCPSCRLRKCYEAGMTLGARKLKKLG
O97775/1-911 AAEGKQKYLCASRNDCTIDKFRRKNCPSCRLRKCYEAGMTLGARKLKKLG
A0A2I2YGE1/1-902 AAEGKQKYLCASRNDCTIDKFRRKNCPSCRLRKCYEAGMTLGARKLKKLG
O97960/1-895 AAEGKQKYLCASRNDCTIDKFRRKNCPSCRLRKCYEAGMTLGARKLKKLG
O97952/1-895 AAEGKQKYLCASRNDCTIDKFRRKNCPSCRLRKCYEAGMTLGARKLKKLG
Q6QT55/1-895 AAEGKQKYLCASRNDCTIDKFRRKNCPSCRLRKCYEAGMTLGARKLKKLG
O97776/1-884 AAEGKQKYLCASRNDCTIDKFRRKNCPSCRLRKCYEAGMTLGARKLKKLG
P15207/1-902 AAEGKQKYLCASRNDCTIDKFRRKNCPSCRLRKCYEAGMTLGARKLKKLG
G1TTV8/1-901 AAEGKQKYLCASRNDCTIDKFRRKNCPSCRLRKCYEAGMTLGARKLKKLG
P19091/1-899 AAEGKQKYLCASRNDCTIDKFRRKNCPSCRLRKCYEAGMTLGARKLKKLG
G1PPI1/1-892 AAEGKQKYLCASRNDCTIDKFRRKNCPSCRLRKCYEAGMTLGARKLKKLG
M3W7I8/1-903 AAEGKQKYLCASRNDCTIDKFRRKNCPSCRLRKCYEAGMTLGARKLKKLG
Q9TT90/1-907 AAEGKQKYLCASRNDCTIDKFRRKNCPSCRLRKCYEAGMTLGARKLKKLG
Q8MIK0/1-912 AAEGKQKYLCASRNDCTIDKFRRKNCPPCRLRKCYEAGMTLGARRLKKLG
A0A3Q7SKY7/1-900 AAEGKQKYLCASRNDCTIDKFRRKNCPSCRLRKCYEAGMTLGARKLKKLG
F6R5G1/1-882 AAEGKQKYLCASRNDCTIDKFRRKNCPSCRLRKCYEAGMTLGARKLKKLG
Q9GKL7/1-896 AAEGKQKYLCASRNDCTIDKFRRKNCPSCRLRKCYEAGMTLGARKLKKLG
U4MHY9/1-894 AAEGKQKYLCASRNDCTIDKFRRKNCPSCRLRKCYEAGMTLGARKLKKLG
S5U7M4/1-890 AAEGKQKYLCASRNDCTIDKFRRKNCPSCRLRKCYEAGMTLGARKLKKLG
F1N2B6/1-890 AAEGKQKYLCASRNDCTIDKFRRKNCPSCRLRKCYEAGMTLGARKLKKLG
A0A2Y9F3J7/1-893 AAEGKQKYLCASRNDCTIDKFRRKNCPSCRLRKCYEAGMTLGARKLKKLG
A0A2Y9M4V8/1-893 AAEGKQKYLCASRNDCTIDKFRRKNCPSCRLRKCYEAGMTLGARKLKKLG
A0A2U3V2H0/1-893 AAEGKQKYLCASRNDCTIDKFRRKNCPSCRLRKCYEAGMTLGARKLKKLG
A0A384ADZ0/1-895 AAEGKQKYLCASRNDCTIDKFRRKNCPSCRLRKCYEAGMTLGARKLKKLG
Q7T1K4/1-777 AAEGNQKYLCASRNDCTIDKFRRKNCPSCRLRKCYEAGMTLGARKLKKLG
Conservation
6
7
9
Quality
Consensus
AAEGKQKYLCASRNDCTIDKFRRKNCPSCRLRKCYEAGMTLGARKLKKLG
Occupancy

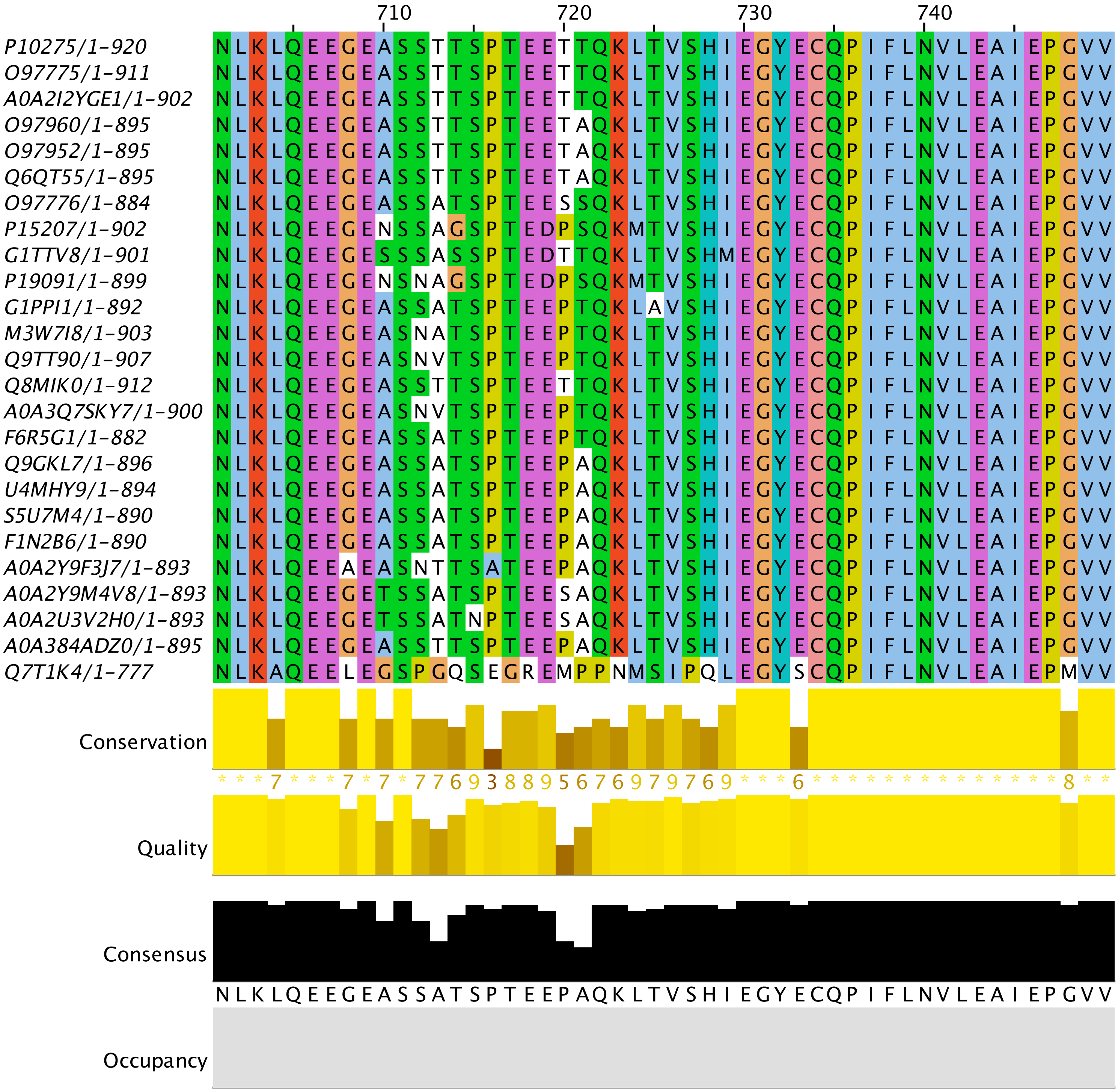

710
720
730
740
P10275/1-920
O97775/1-911
A0A2I2YGE1/1-902
O97960/1-895
O97952/1-895
Q6QT55/1-895
O97776/1-884
P15207/1-902
G1TTV8/1-901
P19091/1-899
G1PPI1/1-892
M3W7I8/1-903
Q9TT90/1-907
Q8MIK0/1-912
A0A3Q7SKY7/1-900
F6R5G1/1-882
Q9GKL7/1-896
U4MHY9/1-894
S5U7M4/1-890
F1N2B6/1-890
A0A2Y9F3J7/1-893
A0A2Y9M4V8/1-893
A0A2U3V2H0/1-893
A0A384ADZ0/1-895
Q7T1K4/1-777
Conservation
Quality
Consensus
NLKLQEEGEASSATSPTEEPAQKLTVSHIEGYECQPIFLNVLEAIEPGVV
Occupancy

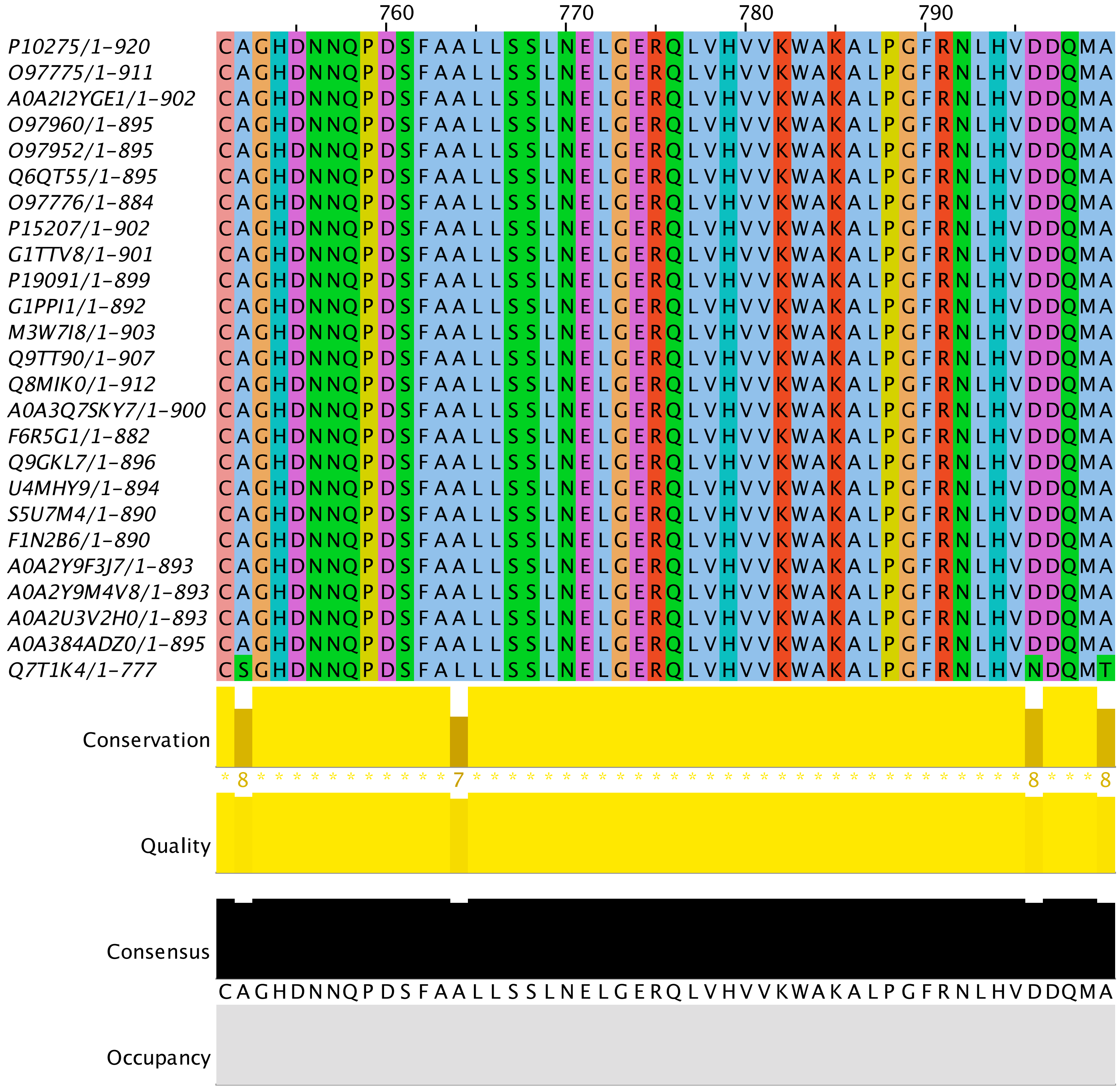

760
770
780
790
P10275/1-920 CAGHDNNQPDSFAALLSSLNELGERQLVHVVKWAKALPGFRNLHVDDQMA
O97775/1-911 CAGHDNNQPDSFAALLSSLNELGERQLVHVVKWAKALPGFRNLHVDDQMA
A0A2I2YGE1/1-902 CAGHDNNQPDSFAALLSSLNELGERQLVHVVKWAKALPGFRNLHVDDQMA
O97960/1-895 CAGHDNNQPDSFAALLSSLNELGERQLVHVVKWAKALPGFRNLHVDDQMA
O97952/1-895 CAGHDNNQPDSFAALLSSLNELGERQLVHVVKWAKALPGFRNLHVDDQMA
Q6QT55/1-895 CAGHDNNQPDSFAALLSSLNELGERQLVHVVKWAKALPGFRNLHVDDQMA
O97776/1-884 CAGHDNNQPDSFAALLSSLNELGERQLVHVVKWAKALPGFRNLHVDDQMA
P15207/1-902 CAGHDNNQPDSFAALLSSLNELGERQLVHVVKWAKALPGFRNLHVDDQMA
G1TTV8/1-901 CAGHDNNQPDSFAALLSSLNELGERQLVHVVKWAKALPGFRNLHVDDQMA
P19091/1-899 CAGHDNNQPDSFAALLSSLNELGERQLVHVVKWAKALPGFRNLHVDDQMA
G1PPI1/1-892 CAGHDNNQPDSFAALLSSLNELGERQLVHVVKWAKALPGFRNLHVDDQMA
M3W7I8/1-903 CAGHDNNQPDSFAALLSSLNELGERQLVHVVKWAKALPGFRNLHVDDQMA
Q9TT90/1-907 CAGHDNNQPDSFAALLSSLNELGERQLVHVVKWAKALPGFRNLHVDDQMA
Q8MIK0/1-912 CAGHDNNQPDSFAALLSSLNELGERQLVHVVKWAKALPGFRNLHVDDQMA
A0A3Q7SKY7/1-900 CAGHDNNQPDSFAALLSSLNELGERQLVHVVKWAKALPGFRNLHVDDQMA
F6R5G1/1-882 CAGHDNNQPDSFAALLSSLNELGERQLVHVVKWAKALPGFRNLHVDDQMA
Q9GKL7/1-896 CAGHDNNQPDSFAALLSSLNELGERQLVHVVKWAKALPGFRNLHVDDQMA
U4MHY9/1-894 CAGHDNNQPDSFAALLSSLNELGERQLVHVVKWAKALPGFRNLHVDDQMA
S5U7M4/1-890 CAGHDNNQPDSFAALLSSLNELGERQLVHVVKWAKALPGFRNLHVDDQMA
F1N2B6/1-890 CAGHDNNQPDSFAALLSSLNELGERQLVHVVKWAKALPGFRNLHVDDQMA
A0A2Y9F3J7/1-893 CAGHDNNQPDSFAALLSSLNELGERQLVHVVKWAKALPGFRNLHVDDQMA
A0A2Y9M4V8/1-893 CAGHDNNQPDSFAALLSSLNELGERQLVHVVKWAKALPGFRNLHVDDQMA
A0A2U3V2H0/1-893 CAGHDNNQPDSFAALLSSLNELGERQLVHVVKWAKALPGFRNLHVDDQMA
A0A384ADZ0/1-895 CAGHDNNQPDSFAALLSSLNELGERQLVHVVKWAKALPGFRNLHVDDQMA
Q7T1K4/1-777 CSGHDNNQPDSFALLLSSLNELGERQLVHVVKWAKALPGFRNLHVNDQMT
Conservation
*8***********7*******************************8***8
Quality
Consensus
CAGHDNNQPDSFAALLSSLNELGERQLVHVVKWAKALPGFRNLHVDDQMA
Occupancy

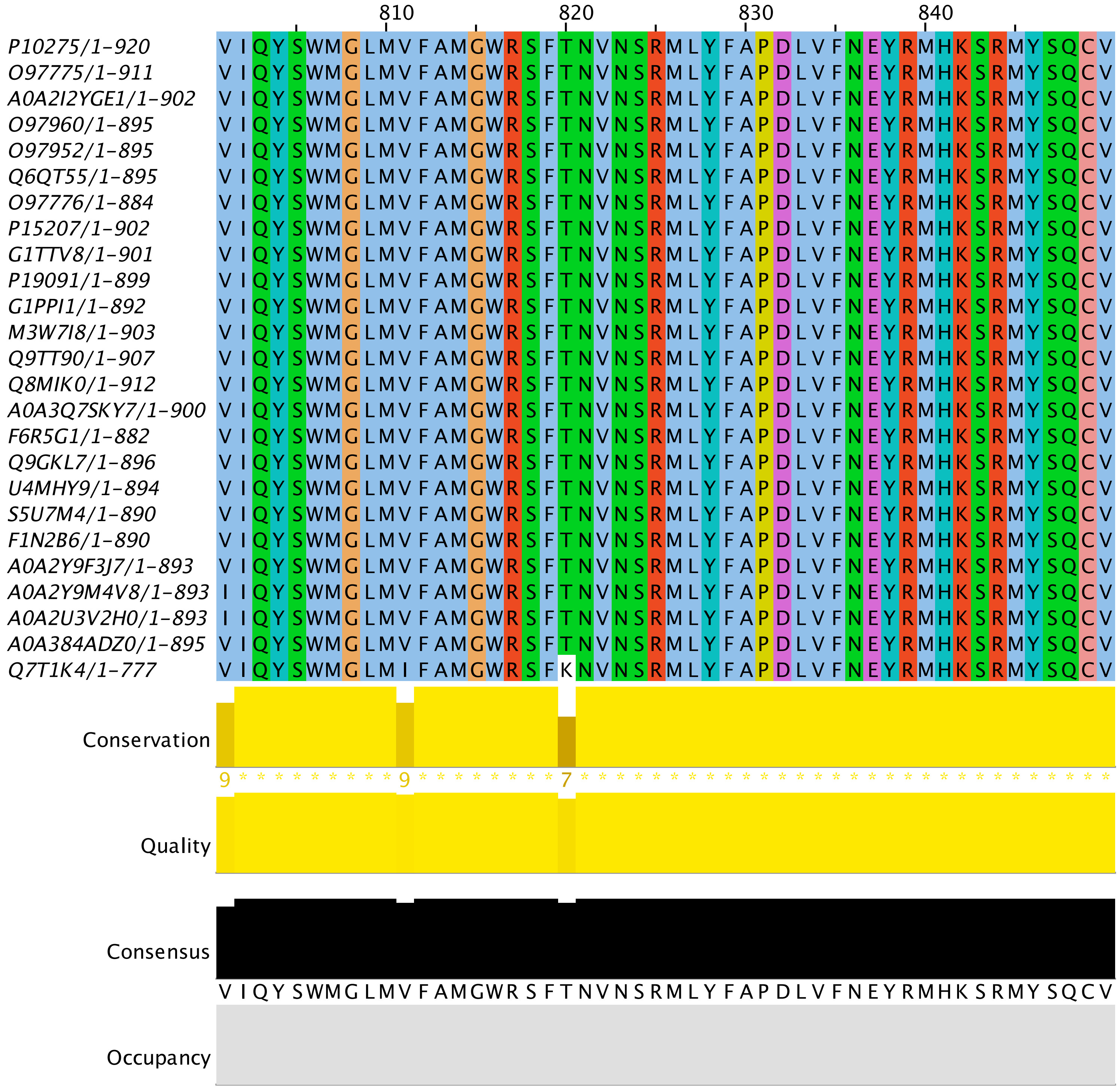
810
820
830
840
P10275/1-920
O97775/1-911
A0A2I2YGE1/1-902
O97960/1-895
O97952/1-895
Q6QT55/1-895
O97776/1-884
P15207/1-902
G1TTV8/1-901
P19091/1-899
G1PPI1/1-892
M3W7I8/1-903
Q9TT90/1-907
Q8MIK0/1-912
A0A3Q7SKY7/1-900
F6R5G1/1-882
Q9GKL7/1-896
U4MHY9/1-894
S5U7M4/1-890
F1N2B6/1-890
A0A2Y9F3J7/1-893
A0A2Y9M4V8/1-893
A0A2U3V2H0/1-893
A0A384ADZ0/1-895
Q7T1K4/1-777
Conservation
Quality
Consensus
VIQYSWMGLMVFAMGWRSFTNVNSRMLYFAPDLVFNEYRMHKSRMYSQCV
Occupancy

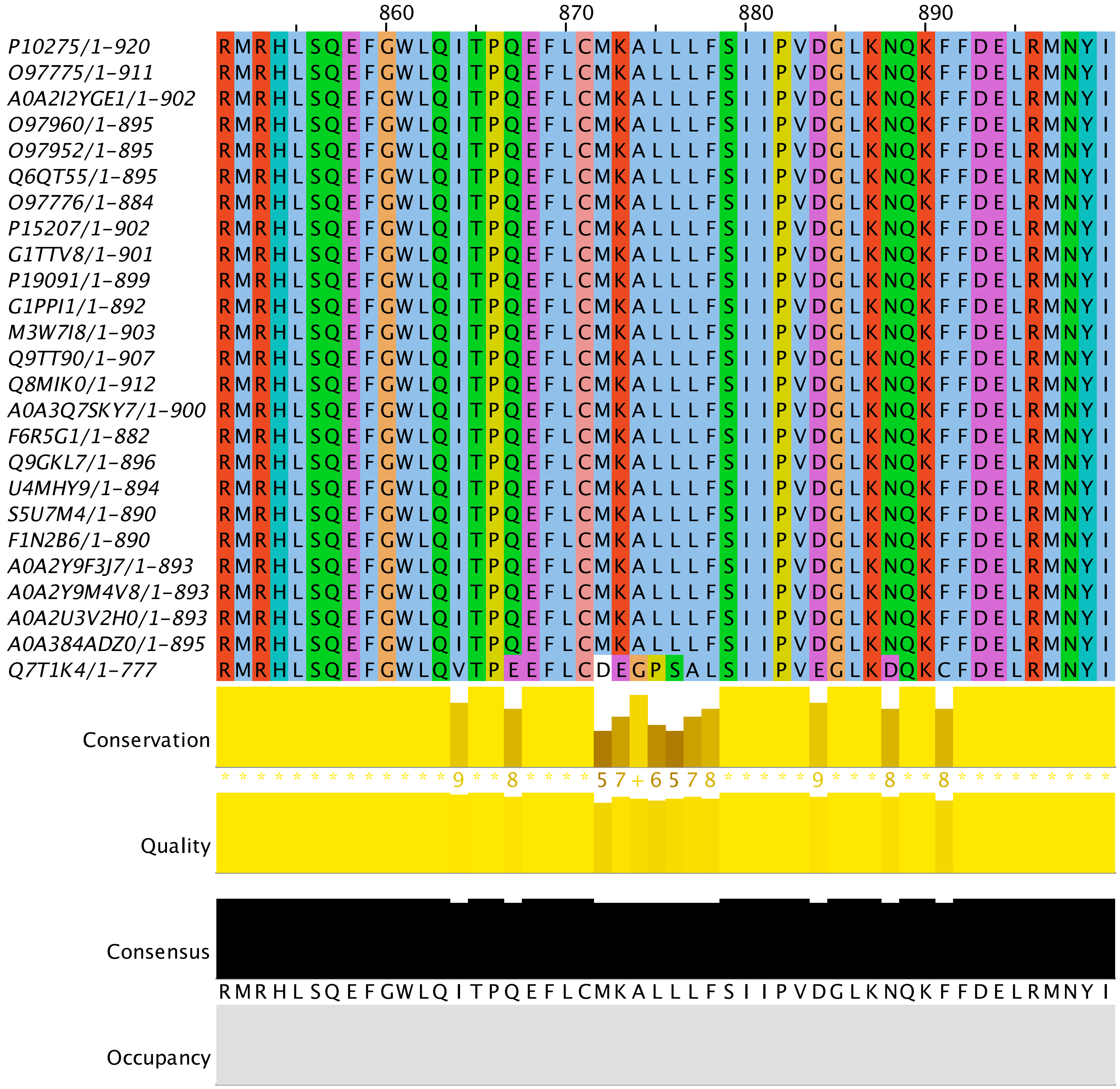

860
870
880
890
P10275/1-920 RMRHLSQEFGWLQITPQEFLCMKALLLFSIIPVDGLKNQKFFDELRMNYI
O97775/1-911 RMRHLSQEFGWLQITPQEFLCMKALLLFSIIPVDGLKNQKFFDELRMNYI
A0A2I2YGE1/1-902 RMRHLSQEFGWLQITPQEFLCMKALLLFSIIPVDGLKNQKFFDELRMNYI
O97960/1-895 RMRHLSQEFGWLQITPQEFLCMKALLLFSIIPVDGLKNQKFFDELRMNYI
O97952/1-895 RMRHLSQEFGWLQITPQEFLCMKALLLFSIIPVDGLKNQKFFDELRMNYI
Q6QT55/1-895 RMRHLSQEFGWLQITPQEFLCMKALLLFSIIPVDGLKNQKFFDELRMNYI
O97776/1-884 RMRHLSQEFGWLQITPQEFLCMKALLLFSIIPVDGLKNQKFFDELRMNYI
P15207/1-902 RMRHLSQEFGWLQITPQEFLCMKALLLFSIIPVDGLKNQKFFDELRMNYI
G1TTV8/1-901 RMRHLSQEFGWLQITPQEFLCMKALLLFSIIPVDGLKNQKFFDELRMNYI
P19091/1-899 RMRHLSQEFGWLQITPQEFLCMKALLLFSIIPVDGLKNQKFFDELRMNYI
G1PPI1/1-892 RMRHLSQEFGWLQITPQEFLCMKALLLFSIIPVDGLKNQKFFDELRMNYI
M3W7I8/1-903 RMRHLSQEFGWLQITPQEFLCMKALLLFSIIPVDGLKNQKFFDELRMNYI
Q9TT90/1-907 RMRHLSQEFGWLQITPQEFLCMKALLLFSIIPVDGLKNQKFFDELRMNYI
Q8MIK0/1-912 RMRHLSQEFGWLQITPQEFLCMKALLLFSIIPVDGLKNQKFFDELRMNYI
A0A3Q7SKY7/1-900 RMRHLSQEFGWLQITPQEFLCMKALLLFSIIPVDGLKNQKFFDELRMNYI
F6R5G1/1-882 RMRHLSQEFGWLQITPQEFLCMKALLLFSIIPVDGLKNQKFFDELRMNYI
Q9GKL7/1-896 RMRHLSQEFGWLQITPQEFLCMKALLLFSIIPVDGLKNQKFFDELRMNYI
U4MHY9/1-894 RMRHLSQEFGWLQITPQEFLCMKALLLFSIIPVDGLKNQKFFDELRMNYI
S5U7M4/1-890 RMRHLSQEFGWLQITPQEFLCMKALLLFSIIPVDGLKNQKFFDELRMNYI
F1N2B6/1-890 RMRHLSQEFGWLQITPQEFLCMKALLLFSIIPVDGLKNQKFFDELRMNYI
A0A2Y9F3J7/1-893 RMRHLSQEFGWLQITPQEFLCMKALLLFSIIPVDGLKNQKFFDELRMNYI
A0A2Y9M4V8/1-893 RMRHLSQEFGWLQITPQEFLCMKALLLFSIIPVDGLKNQKFFDELRMNYI
A0A2U3V2H0/1-893 RMRHLSQEFGWLQITPQEFLCMKALLLFSIIPVDGLKNQKFFDELRMNYI
A0A384ADZ0/1-895 RMRHLSQEFGWLQITPQEFLCMKALLLFSIIPVDGLKNQKFFDELRMNYI
Q7T1K4/1-777 RMRHLSQEFGWLQVTPEEFLCDEGPSALSIIPVEGLKDQKCFDELRMNYI
Conservation
* * * * * * * * * * * * * 9 * * 8 * * * * 5 7 + 6 5 7 8 * * * * * 9 * * * 8 * * 8 * * * * * * * * *
Quality
Consensus
RMRHLSQEFGWLQITPQEFLCMKALLLFSIIPVDGLKNQKFFDELRMNYI
Occupancy

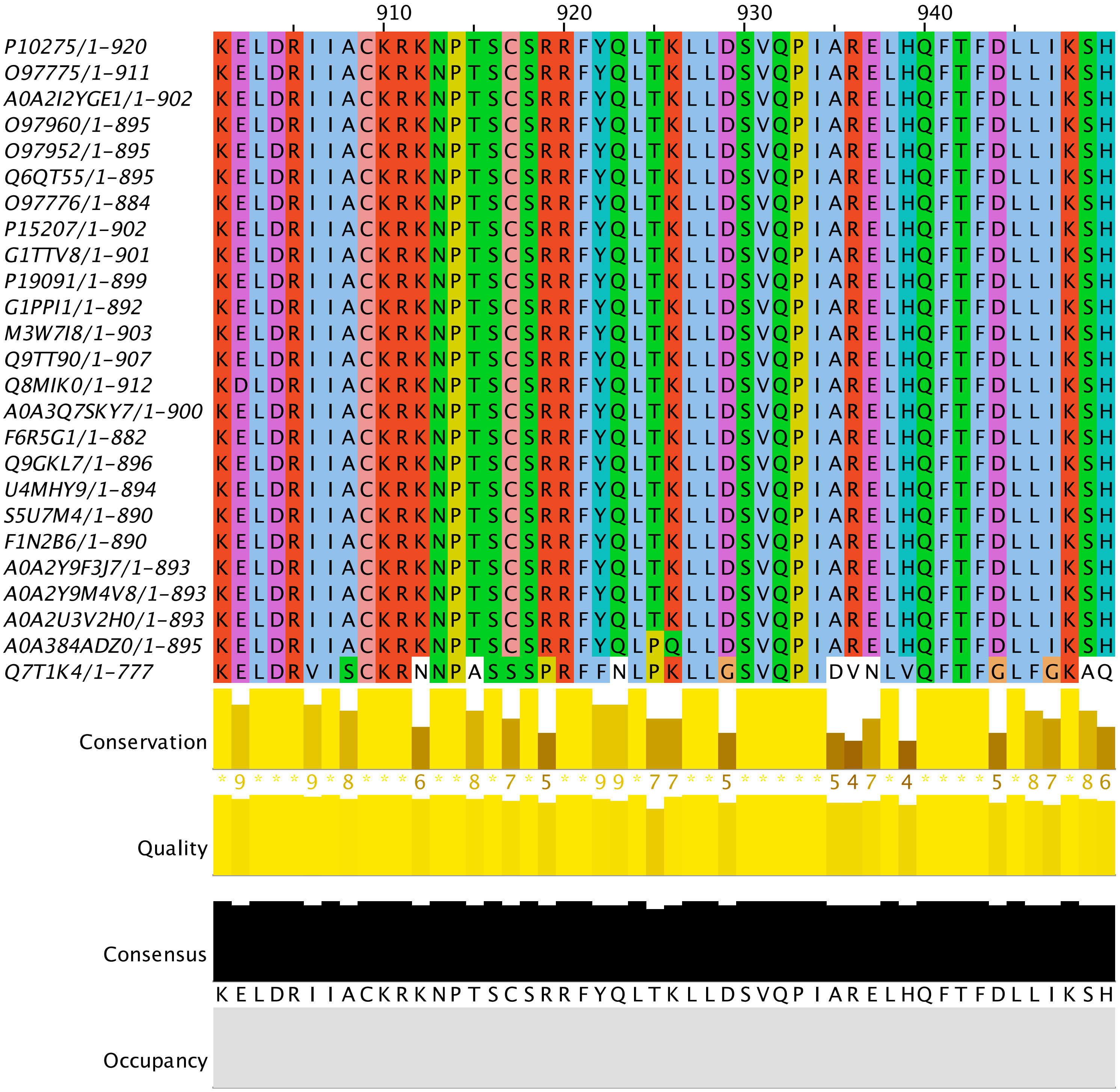
910
920
930
940
P10275/1-920 KELDRIIACKRKNPTSCSRRFYQLTKLLDSVQPIARELHQFTFDLLIKSH
O97775/1-911 KELDRIIACKRKNPTSCSRRFYQLTKLLDSVQPIARELHQFTFDLLIKSH
A0A2I2YGE1/1-902 KELDRIIACKRKNPTSCSRRFYQLTKLLDSVQPIARELHQFTFDLLIKSH
O97960/1-895 KELDRIIACKRKNPTSCSRRFYQLTKLLDSVQPIARELHQFTFDLLIKSH
O97952/1-895 KELDRIIACKRKNPTSCSRRFYQLTKLLDSVQPIARELHQFTFDLLIKSH
Q6QT55/1-895 KELDRIIACKRKNPTSCSRRFYQLTKLLDSVQPIARELHQFTFDLLIKSH
O97776/1-884 KELDRIIACKRKNPTSCSRRFYQLTKLLDSVQPIARELHQFTFDLLIKSH
P15207/1-902 KELDRIIACKRKNPTSCSRRFYQLTKLLDSVQPIARELHQFTFDLLIKSH
G1TTV8/1-901 KELDRIIACKRKNPTSCSRRFYQLTKLLDSVQPIARELHQFTFDLLIKSH
P19091/1-899 KELDRIIACKRKNPTSCSRRFYQLTKLLDSVQPIARELHQFTFDLLIKSH
G1PPI1/1-892 KELDRIIACKRKNPTSCSRRFYQLTKLLDSVQPIARELHQFTFDLLIKSH
M3W7I8/1-903 KELDRIIACKRKNPTSCSRRFYQLTKLLDSVQPIARELHQFTFDLLIKSH
Q9TT90/1-907 KELDRIIACKRKNPTSCSRRFYQLTKLLDSVQPIARELHQFTFDLLIKSH
Q8MIK0/1-912 KDLDRIIACKRKNPTSCSRRFYQLTKLLDSVQPIARELHQFTFDLLIKSH
A0A3Q7SKY7/1-900 KELDRIIACKRKNPTSCSRRFYQLTKLLDSVQPIARELHQFTFDLLIKSH
F6R5G1/1-882 KELDRIIACKRKNPTSCSRRFYQLTKLLDSVQPIARELHQFTFDLLIKSH
Q9GKL7/1-896 KELDRIIACKRKNPTSCSRRFYQLTKLLDSVQPIARELHQFTFDLLIKSH
U4MHY9/1-894 KELDRIIACKRKNPTSCSRRFYQLTKLLDSVQPIARELHQFTFDLLIKSH
S5U7M4/1-890 KELDRIIACKRKNPTSCSRRFYQLTKLLDSVQPIARELHQFTFDLLIKSH
F1N2B6/1-890 KELDRIIACKRKNPTSCSRRFYQLTKLLDSVQPIARELHQFTFDLLIKSH
A0A2Y9F3J7/1-893 KELDRIIACKRKNPTSCSRRFYQLTKLLDSVQPIARELHQFTFDLLIKSH
A0A2Y9M4V8/1-893 KELDRIIACKRKNPTSCSRRFYQLTKLLDSVQPIARELHQFTFDLLIKSH
A0A2U3V2H0/1-893 KELDRIIACKRKNPTSCSRRFYQLTKLLDSVQPIARELHQFTFDLLIKSH
A0A384ADZ0/1-895 KELDRIIACKRKNPTSCSRRFYQLPQLLDSVQPIARELHQFTFDLLIKSH
Q7T1K4/1-777 KELDRVISCKRNNPASSSPRFFNLPKLLGSVQPIDVNLVQFTFGLFGKAQ
Conservation
* 9 * * * 9 * 8 * * * 6 * * 8 * 7 * 5 * * 9 9 * 7 7 * * 5 * * * * * 5 4 7 * 4 * * * * 5 * 8 7 * 8 6
Quality
Consensus
KELDRIIACKRKNPTSCSRRFYQLTKLLDSVQPIARELHQFTFDLLIKSH
Occupancy

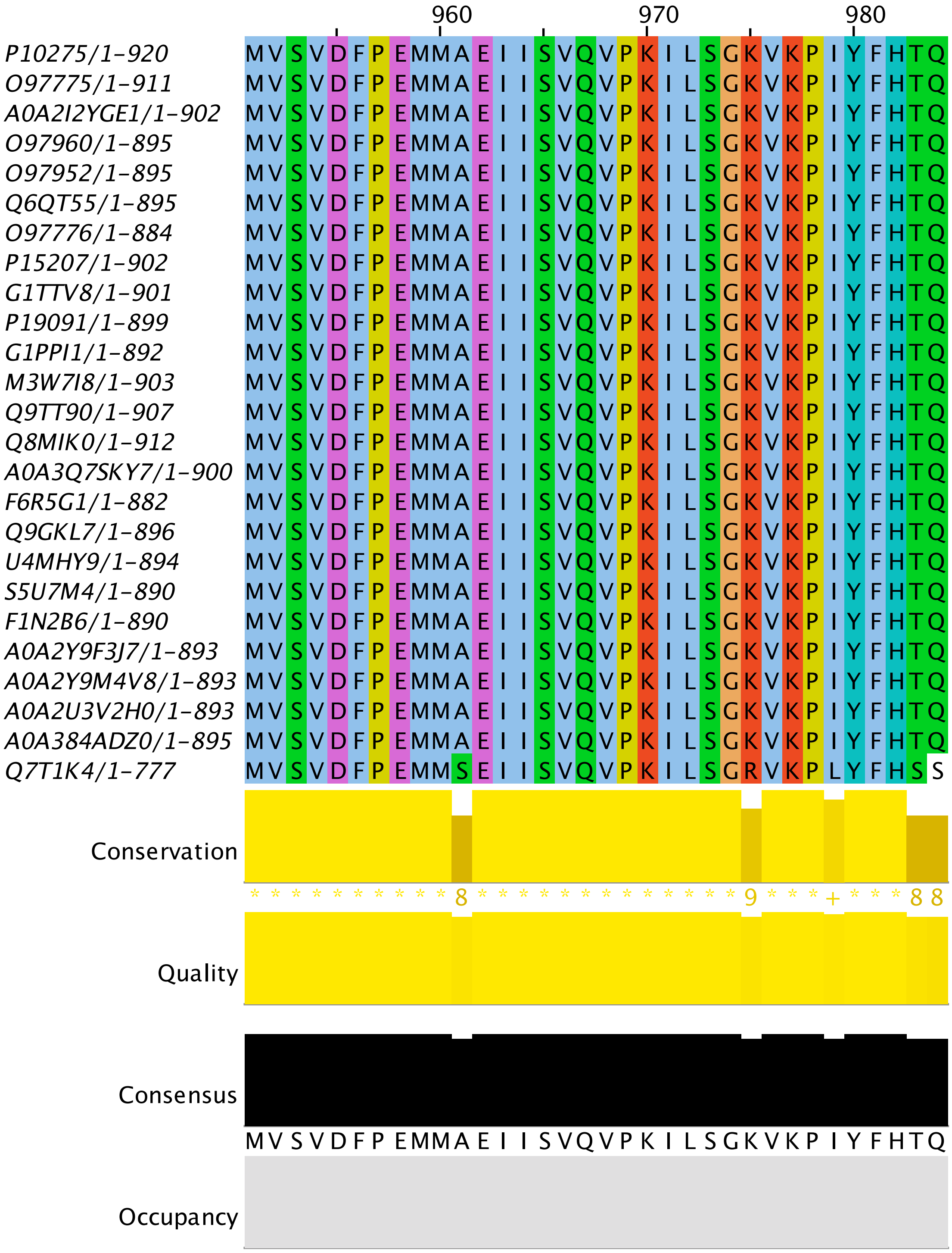

960
970
980
P10275/1-920 MVSVDFPEMMAEIISVQVPKILSGKVKPIYFHTQ
O97775/1-911 MVSVDFPEMMAEIISVQVPKILSGKVKPIYFHTQ
A0A2I2YGE1/1-902 MVSVDFPEMMAEIISVQVPKILSGKVKPIYFHTQ
O97960/1-895 MVSVDFPEMMAEIISVQVPKILSGKVKPIYFHTQ
O97952/1-895 MVSVDFPEMMAEIISVQVPKILSGKVKPIYFHTQ
Q6QT55/1-895 MVSVDFPEMMAEIISVQVPKILSGKVKPIYFHTQ
O97776/1-884 MVSVDFPEMMAEIISVQVPKILSGKVKPIYFHTQ
P15207/1-902 MVSVDFPEMMAEIISVQVPKILSGKVKPIYFHTQ
G1TTV8/1-901 MVSVDFPEMMAEIISVQVPKILSGKVKPIYFHTQ
P19091/1-899 MVSVDFPEMMAEIISVQVPKILSGKVKPIYFHTQ
G1PPI1/1-892 MVSVDFPEMMAEIISVQVPKILSGKVKPIYFHTQ
M3W7I8/1-903 MVSVDFPEMMAEIISVQVPKILSGKVKPIYFHTQ
Q9TT90/1-907 MVSVDFPEMMAEIISVQVPKILSGKVKPIYFHTQ
Q8MIK0/1-912 MVSVDFPEMMAEIISVQVPKILSGKVKPIYFHTQ
A0A3Q7SKY7/1-900 MVSVDFPEMMAEIISVQVPKILSGKVKPIYFHTQ
F6R5G1/1-882 MVSVDFPEMMAEIISVQVPKILSGKVKPIYFHTQ
Q9GKL7/1-896 MVSVDFPEMMAEIISVQVPKILSGKVKPIYFHTQ
U4MHY9/1-894 MVSVDFPEMMAEIISVQVPKILSGKVKPIYFHTQ
S5U7M4/1-890 MVSVDFPEMMAEIISVQVPKILSGKVKPIYFHTQ
F1N2B6/1-890 MVSVDFPEMMAEIISVQVPKILSGKVKPIYFHTQ
A0A2Y9F3J7/1-893 MVSVDFPEMMAEIISVQVPKILSGKVKPIYFHTQ
A0A2Y9M4V8/1-893 MVSVDFPEMMAEIISVQVPKILSGKVKPIYFHTQ
A0A2U3V2H0/1-893 MVSVDFPEMMAEIISVQVPKILSGKVKPIYFHTQ
A0A384ADZ0/1-895 MVSVDFPEMMAEIISVQVPKILSGKVKPIYFHTQ
Q7T1K4/1-777 MVSVDFPEMMSEIISVQVPKILSGRVKPLYFHSS
Conservation
* * * * * * * * * * 8 * * * * * * * * * * * * * 9 * * * + * * * 8 8
Quality
Consensus
MVSVDFPEMMAEIISVQVPKILSGKVKPIYFHTQ
Occupancy
